# Impact of comorbidities on the prognoses of trauma patients: Analysis of a hospital-based trauma registry database

**DOI:** 10.1371/journal.pone.0194749

**Published:** 2018-03-20

**Authors:** Chih-Yuan Wang, Yi-Chan Chen, Ti-Hsuan Chien, Hao-Yu Chang, Yu-Hsien Chen, Chih-Ying Chien, Ting-Shuo Huang

**Affiliations:** 1 Department of General Surgery, Keelung Chang Gung Memorial Hospital, Keelung, Taiwan; 2 Department of Chinese Medicine, College of Medicine, Chang Gung University, Kwei-Shan, Taoyuan, Taiwan; 3 Community Medicine Research Center, Keelung Chang Gung Memorial Hospital, Keelung, Taiwan; National Yang-Ming University, TAIWAN

## Abstract

Here we conducted a retrospective analysis of hospital-based trauma registry database for evaluating the impacts of comorbidities on the prognosis for traumatized patients using Index of Coexistent Comorbidity Disease (ICED) scores. We analyzed the data of patients with blunt trauma who visited emergency department between January 1, 2011, and December 31, 2015 in Chang-Gung Memorial Hospital, Keelung branch, a single level I trauma center in the Northern Taiwan. All consecutive patients with blunt trauma who admitted to the intensive care unit or ordinary ward after initial managements in the emergency department were included. We measured the hospital mortality of blunt traumatized patients using alive discharge as a competing risk. To investigate conditional independence of mortality and ICED scores given Injury Severity Score (ISS), we used log-linear models for modeling independence structures. Overall, we included 4997 patients (median age [IQR], 59 years old (44–75 years); 55.3% male). The mortality rate of blunt traumatized patients was higher in the higher ICED scores group compared to lower ICED scores group (4.7% vs 1.8%, *p* < 0.001). Meanwhile, the higher ICED scores group were associated with older age, higher ISS, and longer hospital stay than lower ICED scores group. Higher ICED group had higher probability of transition-to-death and lower probability of transition-to-discharge under the competing risk model. In the multivariable analysis of transition-specific Cox models, higher ICED group were associated with higher risk for hospital mortality compared to lower ICED group (HR 1.60; [95% CI 1.04–2.47]; *p* = 0.032). Also, higher ICED group were associated with lower probability of transition-to-discharge (HR 0.79; [95%CI 0.73–0.86]; *p* < 0.001). Additionally, higher ICED scores accounted for hospital mortality among patients with ISS < 25. In conclusion, our study suggested that severity of comorbidity was associated with higher hospital mortality among traumatized patients, particularly lower ISS.

## Introduction

Traumatic injury remains a major global public health problem and is associated with massive losses of health and life. According to the World Health Organization (WHO), unintentional injury is the sixth leading cause of death worldwide [1] and similarly, traumatic accidents represented the sixth-leading cause of death in Taiwan in 2016 [2]. Although advances in trauma care systems and vehicle and environmental safety have led to recent decreases in the overall mortality rate associated with trauma, diseases related to pre-existing diseases have increased significantly among elderly trauma patients [3–5].

Societies around the world are facing the issue of population aging. Accordingly, the average age of trauma patients is increasing, although younger patients still comprise the majority of victims [3, 5–7]. In Taiwan, the median age of trauma patient increased from 46 years in 2001 to 60 years in 2014 [2]. Given this trend toward population aging, trauma care systems are now faced with challenges related to the pre-existing comorbidities and impaired physiological reserves of elderly patients.

Several systems for scoring patient injuries are currently available. The Injury Severity Score (ISS) remains the mostly commonly used scoring system for predicting trauma severity and prognosis. The ISS was derived from the Anatomic Injury Score (AIS) but does not include the patient’s age and comorbidity [8]. Although, another predictive system, the Trauma and Injury Severity Score (TRISS), incorporates age, it fails to consider comorbidities [9, 10]. Only the Trauma Risk Adjustment Model (TRAM) incorporates comorbidities [11], however, this system accounts for the number but not the severity of comorbidities. The increasing prevalence of injury and the severity of comorbidities among elderly individuals indicate the need for a scoring system that would consider not only the number but also the severity of comorbidities, as these factors affect traumatic outcomes and thus impact long-term survival [12].

Many clinical scores have been developed to measure or quantify comorbidities. The most prevalent of these scores is the Charlson Index, which includes 19 weighted disease items [13]. Other similar comorbidity measuring scores, such as the Cumulative Illness Rating Scale (CIRS) [14], Geriatric Index of Comorbidity (GIC) [15], Comorbidity-Polypharmacy Score [16], Index of Coexistent Comorbidity Disease (ICED) [17], and Kaplan Index use similar methods to quantify comorbidity. Among these, ICED considers both pre-existing diseases, as well as the overall functional disability caused by comorbidities [18]. Here, we conducted a hospital-based cohort study and competing risk model to investigate the impacts of the ICED scores of trauma patients on hospital outcomes. In addition, we investigated the association between mortality and ICED scores conditional on ISS.

## Materials and methods

### Study design

For this study, we retrospectively analyzed the hospital trauma registry database, which we prospectively registered consecutive trauma patients visiting our emergence department at Chang Gung Memorial hospital, Keelung, a single level I trauma center in North Taiwan, between January 1, 2011 and December 31, 2015. All patients with blunt traumatic injury who were admitted to the intensive care unit or ordinary ward after primary surveillance in the emergency department were enrolled in this study; however, patients with penetrating injuries, burns, out-hospital cardiac arrest (OHCA), an incomplete medical history, an age younger than 20 years, and discharge or death within 24 hours of admission were excluded. We collected the following data for all patients: demographic information, admission duration, vital signs (blood pressure, respiratory rate) and Glasgow coma scale (GCS) at triage, ISS score, number of comorbidities, and discharge condition. All baseline and demographic, including age, sex, GCS, systolic blood pressure (SBP), heart rate (HR), respiratory rate (RR), ISS, history of ICU admission, number of comorbidities, ICED score, and length of hospital stay were recorded. This study was approved by the institutional review board of Chang Gung Memorial Hospital. (IRB approval number:201701589B0D001)

### Assessment of patients' comorbidities

We used the ICED score to evaluate comorbidities [19, 20]. This score is derived from two separate assessments: the Index of Disease Severity (IDS), which comprises 19 medical conditions (each defined by 4 severity levels), and the Index of Physical Impairment (IPI), which comprises 11 physical impairments (each defined by 3 severity levels). The final ICED scores, which range from 0 to 3 in four classes (normal, mild, moderate, severe), are calculated using an algorithm that combines the single highest (“peak”) IDS score with the peak IPI score. In the current study, we defined ICED scores of 0–1 and 2–3 as minor and severe comorbidity, respectively. To ensure the accurate scoring of comorbidities, ICED scores were calculated by two researchers (Ti-Hsuan Chien and Hao-Yu Chang); a third researcher (Yu-Hsien Chen) was consulted to resolve discrepancies.

### Statistical analysis

Continuous variables are presented as medians with interquartile ranges (IQRs) for non-normality, whereas categorical variables are presented as frequencies and percentages. According to the independence assumption in the Cox model, the hazard of the individuals that are censored is equal to the hazard of the individuals that remain in follow-up. In the presence of a competing risk, alive discharge prevents the occurrence of hospital mortality. Thus, the independence assumption is not satisfied. If we had not accounted for competing events, we would have overestimated the cumulative incidence of hospital mortality [21–24]. Thus, we used the competing risk model to investigate the impacts of two levels of ICED scores, with alive discharge as a competing event for hospital mortality.

Additionally, cause-specific Cox models were used to investigate the predictors of two events. The Akaike information criterion (AIC) and substantive knowledge were used for model selection and to identify parsimonious models, respectively. We further investigated the proportional hazards assumption using the modified Schoenfeld residuals test; here, if the proportional hazards assumptions were not met, we investigated the non-linear effects of continuous variables and used time-dependent variables to fit the Cox model [25, 26].

To investigate conditional independence of mortality and ICED scores given ISS, we used log-linear models for modeling independence structures [27] and mosaic plots for bringing them out graphically [28,29]. Mosaic plots have been illustrated in the literature to be an excellent means of visualization for log-linear models to display complete, joint or conditional independence of categorical data. For all of these hypotheses, tables of estimated expected values and residuals (Pearson or deviance) can be computed for hypothesis testing. For inference, the most commonly used aggregation function for the residuals is the sum of squares yielding the associated Pearson or likelihood ratio statistic, respectively [27]. Friendly et al. have showed that residual-based shading scheme can directly be applied to these more complex independence models [28,29]. All confidence intervals (CIs) and tests were two-sided with a significance level of 5%. For the log-linear model, Pearson residuals are standardized deviations of observed from expected values. The heuristic for choosing the cut-off points 2 and 4 is that the Pearson residuals are approximately standard normal distribution, which implies that the highlighted cells are those with residuals individually significant at approximately the alpha = 0.05 and alpha = 0.0001 levels, respectively [30]. All analyses were performed using R software, version 3.4.3 (R Foundation for Statistical Computing, Vienna, Austria) with contributed packages “mstate”, “survival”, and “vcd”. Raw data and computer codes were provided in the supportive information (S1 File, S2 Table, and S2 File)

## Result

A total of 6012 blunt trauma patients were initially selected from our Trauma Registry database. Of these patients, 604 (10%) with ages younger than 20 years and 362 (6%) who died within 24 hours were excluded, as were 43 with missing medical histories and 6 with missing ISSs. Finally, 4997 patients were enrolled in the analysis (S1 Fig). Among them, 4153 (83.1%) were classified as ICED 0 or 1 (i.e., lower ICED), and 844 (16.9%) as ICED 2 or 3 (higher ICED), according to the calculation algorithm. The median age of all patients was 59 years old (IQR: 44–75 years) (S1 Table). Furthermore, 766 (15.3%) patients had ISS scores >15 and 695 (13.9%) were admitted to the ICU.

Table 1 showed the patient’s characteristics stratified by ICED scores. Compared to with the lower ICED group, the higher ICED group tended to include older and female patients with higher ISS, and had a significantly longer hospital stay than the lower ICED group (9 vs. 7 days, p < 0.001). Meanwhile, the higher ICED group also had a significantly higher mortality rate than the lower group (4.7% vs. 1.8%, p < 0.001).

**Table 1:** Patient characteristics stratified by Index of Coexisting Disease scores.

Index of Coexisting Disease category			
Variable	Lower (ICED 0,1) (n = 4153)	Higher (ICED 2,3) (n = 844)	*p* value
Sex, Male (%)	2404 (57.9)	357 (42.3)	< 0.001
Age, median (IQR), years	56 (41, 70)	79 (69, 85)	< 0.001
GCS < 13 (%)	287 (6.9%)	68 (8.1%)	0.268
SBP, median (IQR), mmHg	141 (124, 160)	149 (128, 170)	0.807
ISS, median (IQR)	9 (4, 9)	9 (9, 9)	< 0.001
ISS<16	3534 (85.1)	697 (82.6)	0.129
16≤ISS<25	417 (10.0)	94 (11.1)	
25≤ISS	202 (4.9)	53 (6.3)	
ICU admission (%)	585 (14.1)	110 (13.0)	0.452
Admission days, median (IQR), d	7 (4, 12)	9 (6, 15)	< 0.001
No. of comorbidities (%)			
0	2457 (59.2)	26 (3.1)	< 0.001
1	917 (22.1)	263 (31.2)	
2	627 (15.1)	330 (39.1)	
≥3	152 (3.7)	225 (26.7)	
Death (%)	74 (1.8%)	40 (4.7%)	< 0.001

ICED = Index of Coexisting Disease. IQ = interquartile. GCS = Glasgow coma scale. SBP = systolic blood pressure. ISS = injury severity score. ICU = intensive care unit.

**Fig 1:**
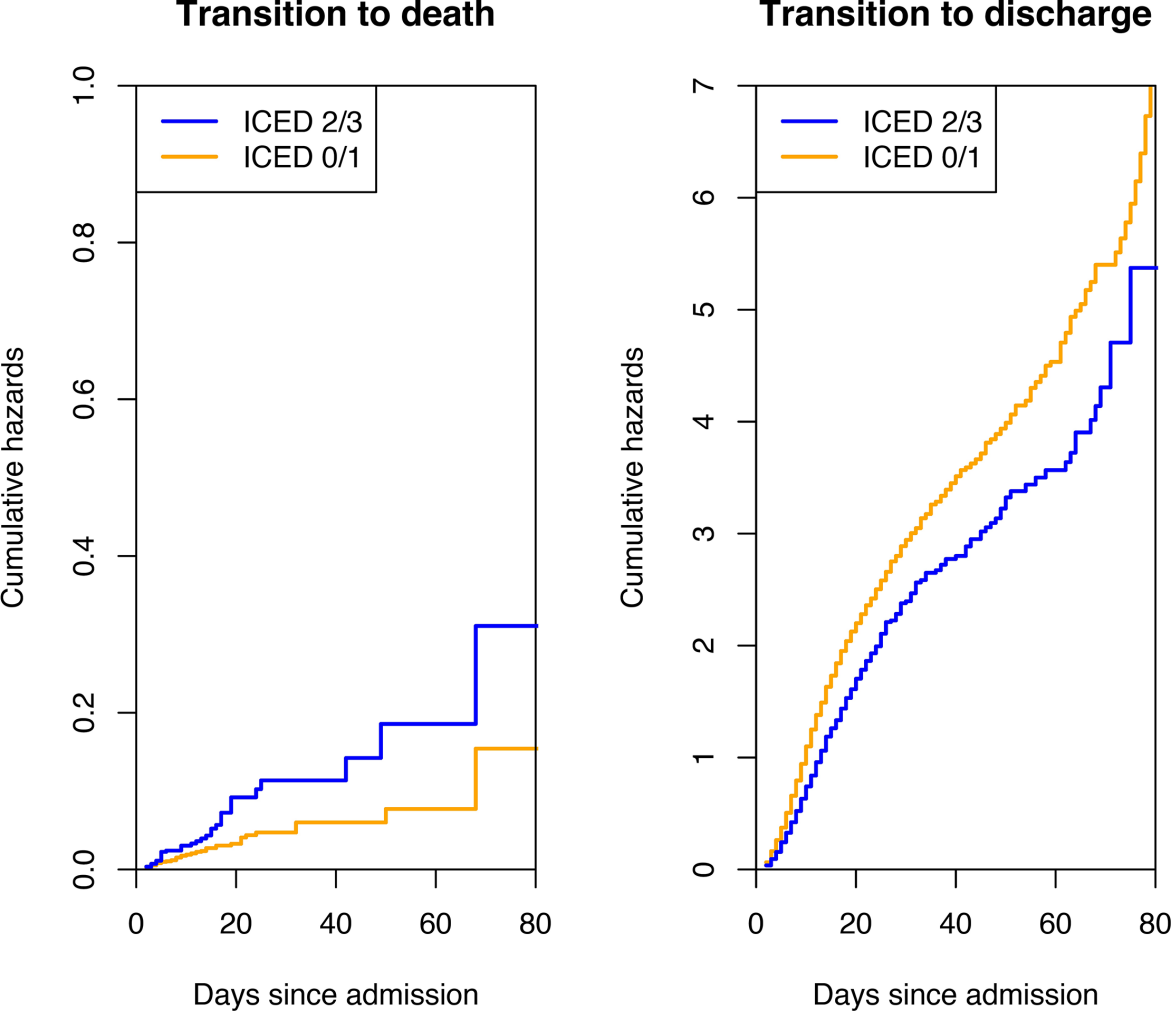
Cumulative hazards stratified by the ICED scores in the competing risk model. Orange lines indicated lower ICED group (n = 4153) whereas blue lines represented higher ICED group (n = 844). Higher ICED group had higher cumulative hazards for transition-to-death and lower cumulative hazards for transition-to-discharge compared with lower ICED group.

**Fig 2:**
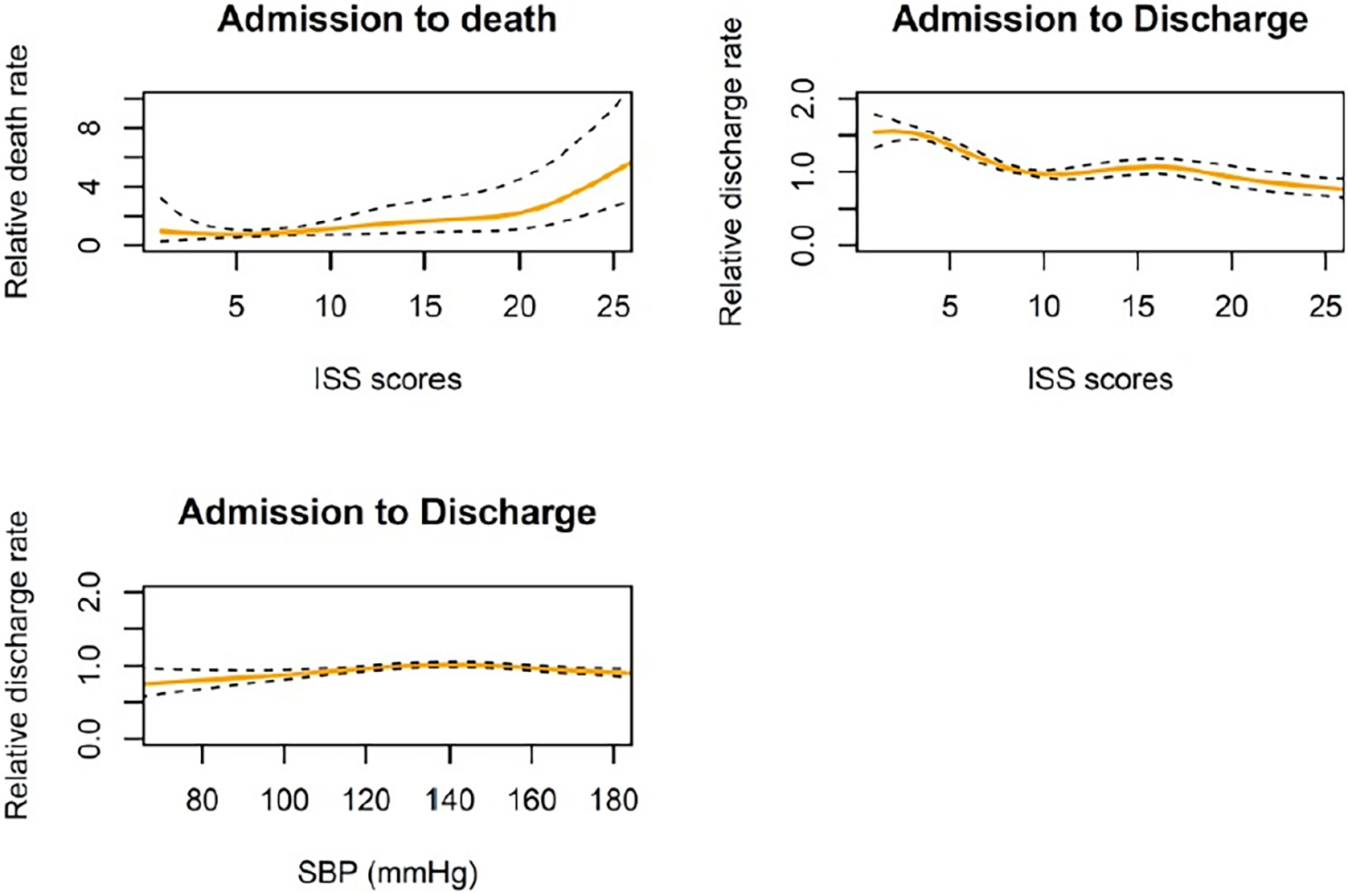
Non-linear effects of injury severity scores on transition-to-death and transition-to-discharge, and non-linear effects of systolic blood pressure on transition-to-discharge. When the ISS reached above 20, the risk for transition-to-death increased exponentially. The p-value of penalized spline for linear effect of ISS score is <0.001, while p-value of penalized spline for non-linear effect of ISS score is 0.018. In the transition-to-discharge, ISS had a linear effect on discharge when ISS<10. The p-value for linear effect of ISS score is <0.001 as well as the nonlinear effect. Lower and higher systolic blood pressure had lower potential for transition-to-discharge. The p-value for linear effect of systolic blood pressure is 0.450, while p-value of for non-linear effect is 0.001. The reference level of injury severity score was 9, while reference level of systolic blood pressure was 150 mmHg.

Fig 1 presents the cumulative hazards for the transition-to-death and transition-to-discharge in the competing risk model, stratified by ICED scores. The higher ICED group had a higher probability of transition-to-death when compared with the lower ICED group (Table 2), while the “transition-to-death” probability in the latter plateaued after 20 days of admission. We next conducted a multivariable analysis using transition-specific Cox models to investigate the predictors of transition-to-death and transition-to-discharge (Table 3). Compared to the lower ICED group, the higher ICED group had a significantly higher transition-to-death risk after adjusting for age, GCS, ICU admission, and ISS (hazard ratio [HR]: 1.60; 95% CI: 1.04–2.47; *p* = 0.032). Notably, an older age, GCS <13, and ICU admission were also significantly associated with a higher risk of transition-to-death, whereas the ISS had a non-linear effect on this outcome (Fig 2). However, the higher ICED group had a lower potential for transition-to-discharge after adjusting for age, sex, GCS, ICU admission, SBP, and ISS (HR: 0.79; 95% CI: 0.73–0.86; *p* < 0.001). An older age, GCS <13, and ICU admission were significantly associated with a lower potential for transition-to-discharge, and ICU admission had a time-dependent effect on this outcome; specifically, comparing to patients without ICU admission, those with ICU admission of <10 days had a HR of 0.39 for transition-to-discharge in the multivariate analysis (*p* < 0.001). And, comparing to patients without ICU admission, those with ICU admission of 10–30 days had a HR of 0.60 for transition-to-discharge (*p* < 0.001). Further, male sex correlated significantly with a lower potential for transition-to-discharge after 10 days of admission. Finally, ISS and SBP had non-linear effects on transition-to-discharge (Fig 2).

**Table 2:** Twenty-, 40-, and 60-day state-occupied probabilities in the competing risk model.

State-occupied probability (95% CI)				
Comorbidity index	Status	20-day	40-day	60-day
ICED 0/1	Admission	9.2 (8.3–10.1)	2.3 (1.8–2.8)	0.8 (0.5–1.1)
ICED 0/1	Death	1.6 (1.2–2.0)	1.8 (1.4–2.2)	1.8 (1.4–2.2)
ICED 0/1	Discharge	89.2 (88.3–90.2)	96.0 (95.3–96.6)	97.4 (97.0–97.9)
ICED 2/3	Admission	15.1 (12.6–17.5)	4.7 (3.2–6.2)	2.0 (1.0–3.0)
ICED 2/3	Death	4.1 (2.7–5.4)	4.3 (2.9–5.7)	4.6 (3.2–6.0)
ICED 2/3	Discharge	80.9 (78.2–83.5)	91.0 (89.0–93.0)	93.5 (91.8–95.2)

ICED = Index of Coexisting Disease. CI = confidence interval.

**Table 3:** Multivariable analysis of transition-specific Cox models.

Variables	Category	HR (95% CI)	*p value*
Transition to death			
Age	10-year increments	1.38 (1.22–1.56)	< 0.001
GCS	Every point increase	0.84 (0.80–0.88)	< 0.001
ICU admission	No	1	
	Yes	3.19 (1.60–6.34)	<0.001
ICED	Lower	1	
	Higher	1.60 (1.04–2.47)	0.032
Transition to discharge			
Age	10-year increments	0.98 (0.96–0.99)	0.008
GCS	Every point increase	1.04 (1.02–1.06)	< 0.001
ICED	Lower	1	
	Higher	0.79 (0.73–0.86)	< 0.001
ICU admission			
< 10 days	Yes vs. No	0.39 (0.33–0.46)	< 0.001
10–30 days	Yes vs. No	0.60 (0.52–0.71)	< 0.001
> 30 days	Yes vs. No	0.88 (0.66–1.18)	0.398
Sex			
< 10 days	Male vs. Female	0.98 (0.91–1.05)	0.493
≥ 10 days	Male vs. Female	0.79 (0.71–0.87)	< 0.001

HR = hazard ratio. CI = confidence interval. ICED = Index of Coexisting Disease. GCS = Glasgow coma scale.

**Fig 3:**
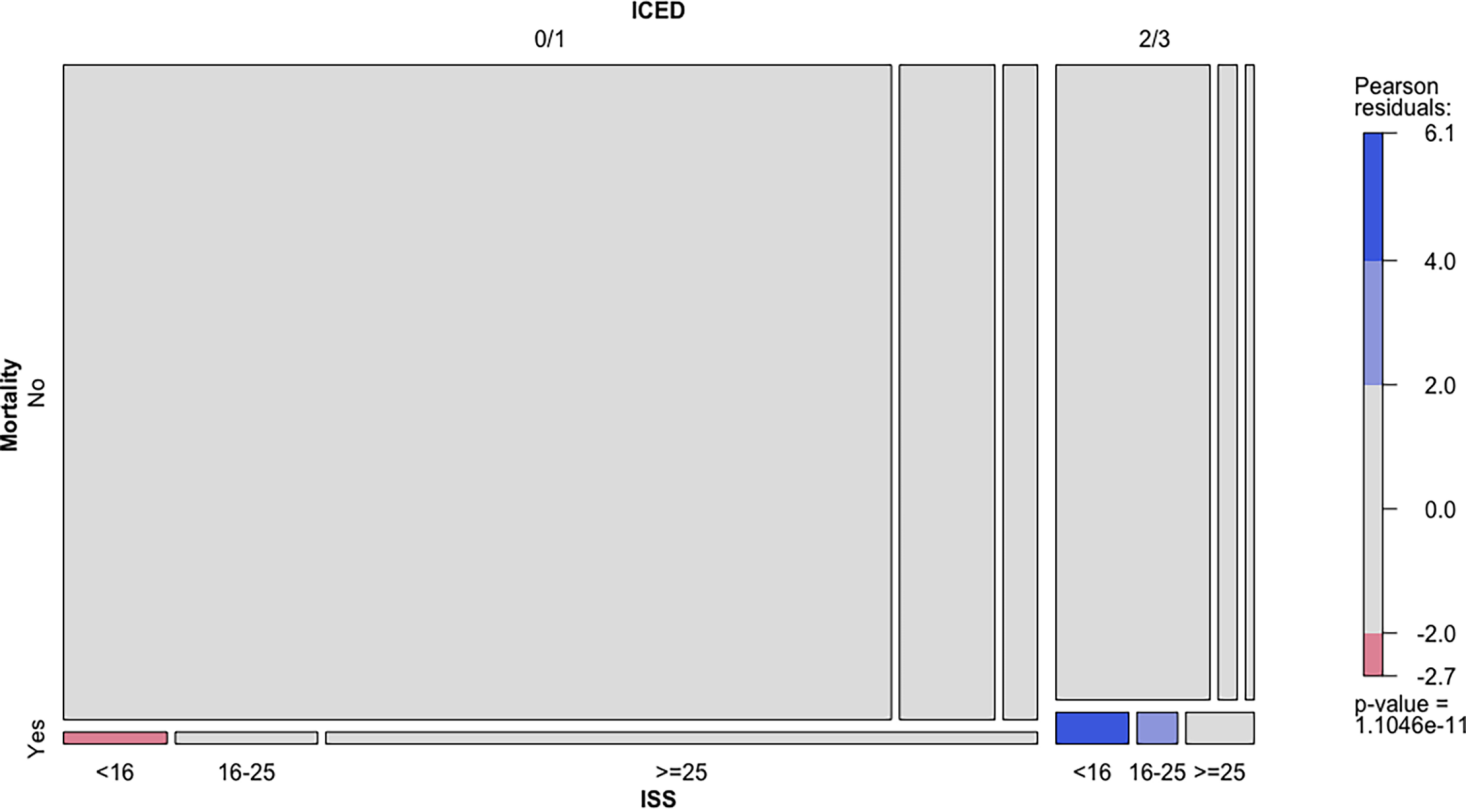
Mosaic plot visualizing the distribution of mortality and ICED scores given ISS using the log-linear model. The size of each cell is proportional to the observed frequency. The Pearson residuals are standardized deviations of observed from expected values. The cut-off points 2 and 4 implies that the highlighted cells are those with residuals individually significant at approximately the alpha = 0.05 and alpha = 0.0001 levels, respectively. Each colored residual violates the null hypotheses of independence. Light blue colored cell indicates positive Pearson residuals at alpha = 0.05 level. Dark blue colored cell indicates positive Pearson residuals at alpha = 0.0001 level. Red colored cell represents negative Pearson residuals at alpha = 0.05 level. The p value is for the log-linear model investigating conditional independence of mortality and ICED scores given ISS, which is highly significant.

The mosaic plot of log-linear model showed that patients with higher ICED score were significantly associated with increased mortality counts than expected (positive Pearson residuals) for trauma patients with ISS<16 and patients with 16≤ISS<25 (Fig 3). Conversely, the cell of lower ICED score and ISS<16 had significant lower mortality counts than the expected value (negative Pearson residuals).

## Discussion

In our study, which incorporated information about comorbidities via the ICED scoring system, we found that an increase in the severity of comorbidities was associated with a poor prognosis among patients with blunt trauma in the competing risk model. In addition, in the log-linear model investigating conditional independence of mortality and ICED scores given ISS, trauma patients with higher ICED scores accounted for mortality among patients with ISS<25, particularly among patients with ISS<16.

Traditionally, the TRISS system [31], which incorporates the ISS for evaluating the severity of anatomic injuries and RTS for evaluating physiologic responses and patient age as a measure of physiologic reserve, has been used to estimate the survival probabilities of trauma patients. Subsequently, Bergeron et al [32] developed the TRISSCOM model, which redefines the age category and adds comorbidities to the TRISS model. The TRISSCOM system includes comorbidity as a binary variable that includes only eight conditions. However, few studies have investigated the effects of comorbidity severity and individual physical activity on trauma outcomes. To the best of our knowledge, our study is the first one to clarify these impacts.

In addition to the paucity of general information about the impacts of comorbidities, no standard or uniform method existed for the evaluation or quantification of comorbidity severity in elderly traumatized patients. Recently, some studies investigated whether incorporating comorbidity and polypharmacy data would improve abilities to predict the mortality outcomes of elderly trauma patients [15, 16, 18, 33]. In addition, the TRAM, developed by Lynne et al [11], incorporates the number of comorbidities and thus yields a superior prediction performance, compared to the TRISS model. Still, although TRAM improved prediction accuracy, it failed to account for comorbidity severity. In the current study, therefore, we used ICED scores as a measure of comorbidity severity and physical impairments in our patient sample. As noted previously, a patient’s physiologic reserve and comorbidity-related responses, rather than age or the number of comorbidities, were found to associate with outcomes [34, 35]. Future studies are recommended to investigate predicting performance incorporating different comorbidity scoring systems, and to create new coding algorithms specific for trauma patients.

The commonly used TRISS system dichotomizes patient age at 55 years. However, this approach compromised the validity of outcome predictions in an aging population [36]. This is a significant concern because advances in medical and surgical care are expected to increase the average human lifespan. Accordingly, we are approaching a "geriatric era", wherein the field of traumatology will gradually transition to a geriatric science [3]. For example, in the Major Trauma Outcome Study (MTOS) of 1987, trauma patients older than 55 years accounted for only 15.5% of the sample [10], whereas in the current study, half of the trauma patients were older than 55 years, and one-third were older than 70 years. We note that this increase in the age of the trauma population also occurred in other studies, despite differences in the definitions of elderly patients [37–41]. However, it seems impractical to define a clear age cut-off because differences in comorbidities will lead to differences in actual physiological changes and the ability to recover from major trauma, even among patients of the same age [42].

In the present study, the observed association of a higher ICED score with an older age is straightforward, as elderly patients tend to have more comorbidities and physical impairments. We also note that patients in the higher ICED group also had relatively higher ISSs; however, there was not statistically significant stratified by ISSs. In addition, ICU admission was not statistically significant between 2 groups. The possible reason might be our inclusion criteria. We included patients surviving more than 24 hours of admission. Patients with higher ICED and ISSs might suffer from unfavorable outcomes earlier.

For our investigation of prognostic factors, we selected a competing risk model that included nonlinear and time-dependent effects for several reasons. First, most studies of the impacts of comorbidities on trauma patients used a logistic regression model [12, 33, 43] that only reflected the risk factors for transition-to-death. However, a single variable might have different effects on different transitions in the presence of competing risks. Second, transition-specific Cox models can be used to investigate time-dependent effects when the proportional hazards assumption is not hold. In addition, this approach allowed us to calculate the state-occupied probability during the period of hospital admission, which better reflected the real-world situation over time [44]. In the “transition to death” situation, patients with higher ICED scores had higher mortality throughout the admission period, whereas all mortality among the lower ICED group occurred within 20 days. These results suggest that early mortality in the lower ICED group could be attributed to a higher ISS, whereas both early and late mortality in the higher ICED group were influenced by the ISS and comorbidities. Despite improvements in early mortality (e.g., exsanguination), the rate of late mortality, or events occurring beyond 1 week after trauma, remained unchanged [45]. In one study reporting a late mortality rate of 2.36%, 38.69% of these cases involved victims older than 71 years [46]. These late mortality events often involve sepsis and multiorgan failure associated with the complications of trauma and the patient’s comorbidities. These resuts are consistent with our log-linear model that higher ICED score accounted for mortality for patients with lower ISS.

In addition to comorbidity, several known risk factors associated with admission-to-death and admission-to-discharge were identified. In contrast to other studies [47, 48], we observed non-linear relationships of ISS with “admission to death” and “admission to discharge”. The relationship of SBP with the transition-to-discharge transition was also non-linear. Interestingly, we observed a significant association of the male sex with a lower potential for transition-to-discharge. As we also observed significant associations of the male sex with a lower GCS, increased likelihood of ICU admission, and lower comorbidity scores, the sex-based effects might be driven by a combination of all these factors.

The present study had several limitations of note. First, a higher ICED score was found to associate with age, sex, and ISS. Therefore, we could not exclude the effects of these potential confounders on comorbidities. Second, we did not thoroughly compare all comorbidity scoring systems into the prediction models. Indeed, there is no consensus exists regarding the coding of comorbidities in traumatized patients. Therefore, further studies of this topic are warranted. In addition, comparing different comorbidity scoring systems in the prediction performance for traumatized patients is important as well. Finally, our study was conducted at a single-center, which might influence the external validity and generalizability of our findings.

## Conclusion

Our study suggested that severity of comorbidity was associated with higher hospital mortality among traumatized patients. In addition, higher ICED scores accounted for mortality among traumatized patients with ISS<25, particularly among patients with ISS<16.

## Supporting information

S1 Fig Flow chart of the patient selection process. (TIF)

S1 File (CSV)

S2 File (PDF)

S1 Table Baseline patient demographics and characteristics. (DOCX)

S2 Table Data description. (DOCX)
