# Supplementary figures and images for: Impact of comorbidities on the prognoses of trauma patients: Analysis of a hospital-based trauma registry database
Source: PLoS One. 2018 Mar 20;13(3):e0194749. doi: 10.1371/journal.pone.0194749 (PMC5860791; doi:10.1371/journal.pone.0194749)

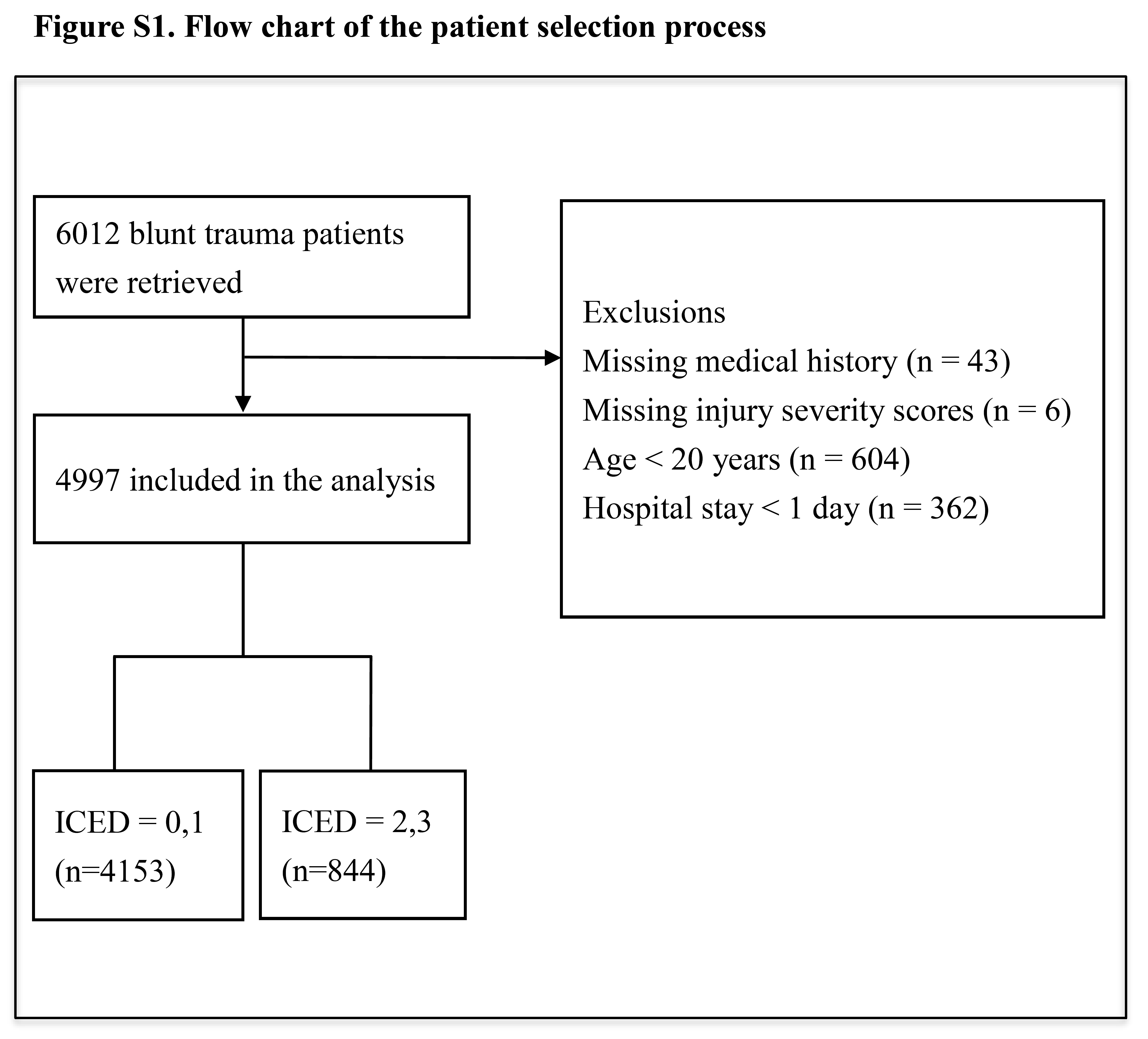

Supplement: S1 Fig — (TIF) [file pone.0194749.s001.tif]
