# Supplementary material for: Impact of comorbidities on the prognoses of trauma patients: Analysis of a hospital-based trauma registry database
Source: PLoS One. 2018 Mar 20;13(3):e0194749. doi: 10.1371/journal.pone.0194749 (PMC5860791; doi:10.1371/journal.pone.0194749)
Supplement: S2 File — (PDF) [file pone.0194749.s003.pdf]

Comorbidity of Trauma

Ting-Shuo Huang

2017/02/28

Load required packages

```
library(doBy)
library(gmodels)
library(tableone)
library(colorspace)
library(survival)
library(mvna)
library(etm)
library(mstate)
library(lattice)
library(rms)
```

```
## Loading required package: Hmisc

## Loading required package: Formula

## Loading required package: ggplot2

##
## Attaching package: 'Hmisc'

## The following objects are masked from 'package:base':
##
##     format.pval, units

## Loading required package: SparseM

##
## Attaching package: 'SparseM'

## The following object is masked from 'package:base':
##
##     backsolve
```

```
library(ggplot2)
library(magrittr)
```

Patient selection and prepare datasets (S1 Figure)

```
#Read data of 2015
trauma2015<-read.csv("Trauma2015d.csv")
##N=1402
#summary(trauma2015)
##Retrieve blunt trauma patient(1402-1285=117 excluding)
trauma2015=subset(trauma2015, mechanism=="Blunt")
##N=1285
```

```
#summary(trauma2015)
##Retrieve patients with ICED=A,B(1285-1243=42 excluding)
trauma2015=subset(trauma2015, ICED_23=="A"|ICED_23=="B")
##N=1243
#summary(trauma2015)
##Exclude missing data of ISS (1243-1237=6)
trauma2015=subset(trauma2015, !is.na(iss))
##N=1237
##Exclude missing data of Co_no (1237-1236=1)
trauma2015=subset(trauma2015, !is.na(Co_no))
##N=1236
#summary(trauma2015)
table(trauma2015$Discharge)
```

```
##
##              AAD      CriticalAAD            Death DischargeChronic
##               17                8               26               12
##  DischargeHealth      ERdischarge    TransferAcute  TransferChronic
##             1064               77                4               28
```

```
##Read data of 2011-2014
Trauma20112014<-read.csv("Trauma20112014d.csv")
##N=4727
table(Trauma20112014$Discharge)
```

```
##
##                    AAD               CriticalAAD                     Death
##                     70                        57                       103
##       DischargeChronic           DischargeHealth             TransferAcute
##                    101                      4341                         9
##        TransferChronic TrnasferotherCommittee
##                     22                        24
```

```
##Combine 2 datasets
trauma=rbind(trauma2015,Trauma20112014)
##N==5963
##Retrieve age>=20 y/o, 5963-5359=604 excluding
traumaAGE<-subset(trauma, age>=20)
##N=5359
##Exclude death within 24 hours (5359-4997=362)
traumaAD<-subset(traumaAGE, ADday>1)
##Coding death and alive discharge
##0=censoring, 1=death, 2=alive discharge 114/4997=2.28%
#table(traumaAD$Discharge)
traumaAD$status=ifelse(traumaAD$Discharge=="CriticalAAD" | traumaAD$Discharge=="Death", 1, ifelse(trauma
table(traumaAD$status)
```

```
##
##    0    1    2
##   73  114 4810
```

```
##Coding age group
traumaAD$AGE=ifelse(traumaAD$age <=60, 1, ifelse(traumaAD$age>60&traumaAD$age<=70 , 2, ifelse(traumaAD$a
#table(traumaAD$AGE)
##Coding respiratpry rate
traumaAD$RR=ifelse(traumaAD$rr <=14, 1, ifelse(traumaAD$rr>14&traumaAD$rr<=24 ,2, 3))
```

```
#table(traumaAD$RR)

##Coding shock
traumaAD$SBP=ifelse(traumaAD$sbp <90, 1, 0)
#table(traumaAD$SBP)

##Coding GCS
traumaAD$GCS=ifelse(traumaAD$gcs <13,0,1)
#table(traumaAD$GCS)

##Coding ISS
traumaAD$ISS_3=ifelse(traumaAD$iss <16, 1, ifelse(traumaAD$iss>=16&traumaAD$iss<25 ,2, 3))
#table(traumaAD$ISS_3)

##Coding  admission
traumaAD$ICU=ifelse(traumaAD$LeaveER=="ICU", 1, 0)
#table(traumaAD$ICU)

##Prepare dataset
trauma=subset(traumaAD,select=c( "Sex", "age","alcohol","gcs", "GCS", "pr", "sbp", "rr",  "AIS", "AIS_1

#summary(trauma)

trauma$GCS=as.factor(trauma$GCS)
#summary(trauma$GCS)

trauma$ISS_3=as.factor(trauma$ISS_3)
#summary(trauma$ISS_3)

trauma$ICU=as.factor(trauma$ICU)
#summary(trauma$ICU)
```

Data exploration and create S1 Table and Table 1

```
#CreateTableOne(data = trauma)
dput(names(trauma))
```

```
## c("Sex", "age", "alcohol", "gcs", "GCS", "pr", "sbp", "rr", "AIS",
## "AIS_1", "AIS_2", "iss", "NISS", "ICU", "ADday", "death", "Co_no",
## "ICED_N", "ICED_23", "status", "ISS_3")
```

```
trauma$Co.no=ifelse(trauma$Co_no==0, 1, ifelse(trauma$Co_no==1, 2, ifelse(trauma$Co_no==2, 3, 4)))
#table(trauma$Co_no)
#table(trauma$Co.no)
#str(trauma$Co.no)
trauma$Co.no=as.factor(trauma$Co.no)

## Vector of categorical variables that need transformation
catVars <- c( "Sex", "AGE", "alcohol", "GCS",
 "ISS_3",  "death","ICU", "Co_no","Co.no", "ICED_N", "ICED_23", "status")

myVars=c("Sex", "age", "AGE", "alcohol", "gcs", "GCS",
```

```r
"pr", "sbp",  "rr",  "iss", "ISS_3", "NISS", "ICU", "ADday", "death", "Co_no", "ICED_N", "ICED_23",
"status", "Co.no")
## Create a TableOne object
tab2<-CreateTableOne(vars = myVars, data = trauma, factorVars = catVars)
```

```
## Warning in ModuleReturnVarsExist(vars, data): The data frame does not have:
## AGE Dropped
```

```
## Warning in ModuleReturnVarsExist(factorVars, data): The data frame does not
## have: AGE Dropped
```

```r
print(tab2, showAllLevels = TRUE)
```

```
##
##                       level Overall
##    n                          4997
##    Sex (%)            F       2236 (44.7)
##                       M       2761 (55.3)
##    age (mean (sd))           58.49 (19.70)
##    alcohol (%)        0       4674 (93.5)
##                       1        323 ( 6.5)
##    gcs (mean (sd))           14.44 (2.01)
##    GCS (%)            0        355 ( 7.1)
##                       1       4642 (92.9)
##    pr (mean (sd))            86.08 (17.89)
##    sbp (mean (sd))          144.42 (29.39)
##    rr (mean (sd))            18.45 (1.74)
##    iss (mean (sd))            8.88 (6.36)
##    ISS_3 (%)          1       4231 (84.7)
##                       2        511 (10.2)
##                       3        255 ( 5.1)
##    NISS (mean (sd))          10.01 (7.30)
##    ICU (%)            0       4302 (86.1)
##                       1        695 (13.9)
##    ADday (mean (sd))         10.49 (10.18)
##    death (%)          0       4880 (97.7)
##                       1        114 ( 2.3)
##    Co_no (%)          0       2483 (49.7)
##                       1       1180 (23.6)
##                       2        957 (19.2)
##                       3        240 ( 4.8)
##                       4         99 ( 2.0)
##                       5         28 ( 0.6)
##                       6          7 ( 0.1)
##                       7          2 ( 0.0)
##                       8          1 ( 0.0)
##    ICED_N (%)         0       2302 (46.1)
##                       1       1850 (37.0)
##                       2        646 (12.9)
##                       3        199 ( 4.0)
##    ICED_23 (%)        A       4153 (83.1)
##                       B        844 (16.9)
##    status (%)         0         73 ( 1.5)
##                       1        114 ( 2.3)
##                       2       4810 (96.3)
```

```
##    Co.no (%)          1       2483 (49.7)
##                       2       1180 (23.6)
##                       3        957 (19.2)
##                       4        377 ( 7.5)
```

```
biomarkers <- c("age", "gcs", "pr", "sbp", "rr","iss", "NISS", "ADday", "Co_no")
tab3 <- CreateTableOne(vars = myVars, strata = "ICED_23" , data = trauma, factorVars = catVars)
```

```
## Warning in ModuleReturnVarsExist(vars, data): The data frame does not have:
## AGE Dropped

## Warning in ModuleReturnVarsExist(vars, data): The data frame does not have:
## AGE Dropped
```

```
##print(tab3, showAllLevels = TRUE)
##Table 1
print(tab3, nonnormal = biomarkers, exact = "stage", smd = TRUE)
```

```
##                       Stratified by ICED_23
##                         A                        B
##    n                     4153                     844
##    Sex = M (%)           2404 (57.9)              357 ( 42.3)
##    age (median [IQR])   56.00 [41.00, 70.00]    79.00 [69.00, 85.00]
##    alcohol = 1 (%)       298 ( 7.2)               25 (  3.0)
##    gcs (median [IQR])   15.00 [15.00, 15.00]    15.00 [15.00, 15.00]
##    GCS = 1 (%)           3866 (93.1)              776 ( 91.9)
##    pr (median [IQR])    84.00 [74.00, 96.00]    84.00 [74.00, 97.00]
##    sbp (median [IQR])  141.00 [124.00, 160.00] 149.00 [128.00, 170.25]
##    rr (median [IQR])    18.00 [18.00, 20.00]    18.00 [18.00, 20.00]
##    iss (median [IQR])    9.00 [4.00, 9.00]       9.00 [9.00, 9.00]
##    ISS_3 (%)
##       1                 3534 (85.1)              697 ( 82.6)
##       2                  417 (10.0)               94 ( 11.1)
##       3                  202 ( 4.9)               53 (  6.3)
##    NISS (median [IQR])   9.00 [4.00, 12.00]      9.00 [9.00, 11.25]
##    ICU = 1 (%)           585 (14.1)              110 ( 13.0)
##    ADday (median [IQR])  7.00 [4.00, 12.00]      9.00 [6.00, 15.00]
##    death = 1 (%)          74 ( 1.8)               40 (  4.7)
##    Co_no (%)
##       0                 2457 (59.2)               26 (  3.1)
##       1                  917 (22.1)              263 ( 31.2)
##       2                  627 (15.1)              330 ( 39.1)
##       3                  102 ( 2.5)              138 ( 16.4)
##       4                   36 ( 0.9)               63 (  7.5)
##       5                    9 ( 0.2)               19 (  2.3)
##       6                    3 ( 0.1)                4 (  0.5)
##       7                    1 ( 0.0)                1 (  0.1)
##       8                    1 ( 0.0)                0 (  0.0)
##    ICED_N (%)
##       0                 2302 (55.4)                0 (  0.0)
##       1                 1850 (44.5)                0 (  0.0)
##       2                    1 ( 0.0)              645 ( 76.4)
##       3                    0 ( 0.0)              199 ( 23.6)
##    ICED_23 = B (%)         0 ( 0.0)              844 (100.0)
##    status (%)
```

```
##     0                     57 ( 1.4)             16 (  1.9)
##     1                     74 ( 1.8)             40 (  4.7)
##     2                   4022 (96.8)            788 ( 93.4)
##   Co.no (%)
##     1                   2457 (59.2)             26 (  3.1)
##     2                    917 (22.1)            263 ( 31.2)
##     3                    627 (15.1)            330 ( 39.1)
##     4                    152 ( 3.7)            225 ( 26.7)
##                        Stratified by ICED_23
##                         p      test    SMD
##   n
##   Sex = M (%)           <0.001          0.316
##   age (median [IQR])    <0.001 nonnorm  1.313
##   alcohol = 1 (%)       <0.001          0.193
##   gcs (median [IQR])     0.003 nonnorm  0.012
##   GCS = 1 (%)            0.268          0.044
##   pr (median [IQR])      0.381 nonnorm  0.050
##   sbp (median [IQR])    <0.001 nonnorm  0.205
##   rr (median [IQR])      0.911 nonnorm  0.024
##   iss (median [IQR])    <0.001 nonnorm  0.207
##   ISS_3 (%)              0.129          0.074
##     1
##     2
##     3
##   NISS (median [IQR])   <0.001 nonnorm  0.118
##   ICU = 1 (%)            0.452          0.031
##   ADday (median [IQR])  <0.001 nonnorm  0.259
##   death = 1 (%)         <0.001          0.167
##   Co_no (%)             <0.001          1.637
##     0
##     1
##     2
##     3
##     4
##     5
##     6
##     7
##     8
##   ICED_N (%)            <0.001          91.130
##     0
##     1
##     2
##     3
##   ICED_23 = B (%)       <0.001          NaN
##   status (%)            <0.001          0.173
##     0
##     1
##     2
##   Co.no (%)             <0.001          1.636
##     1
##     2
##     3
##     4
```

```r
summary(trauma$age)
```

```
##    Min. 1st Qu.  Median    Mean 3rd Qu.    Max.
##   20.00   44.00   59.00   58.49   75.00  104.00
```

```r
par(mfrow=c(1,1))
boxplot(iss~Sex, data=trauma)
```

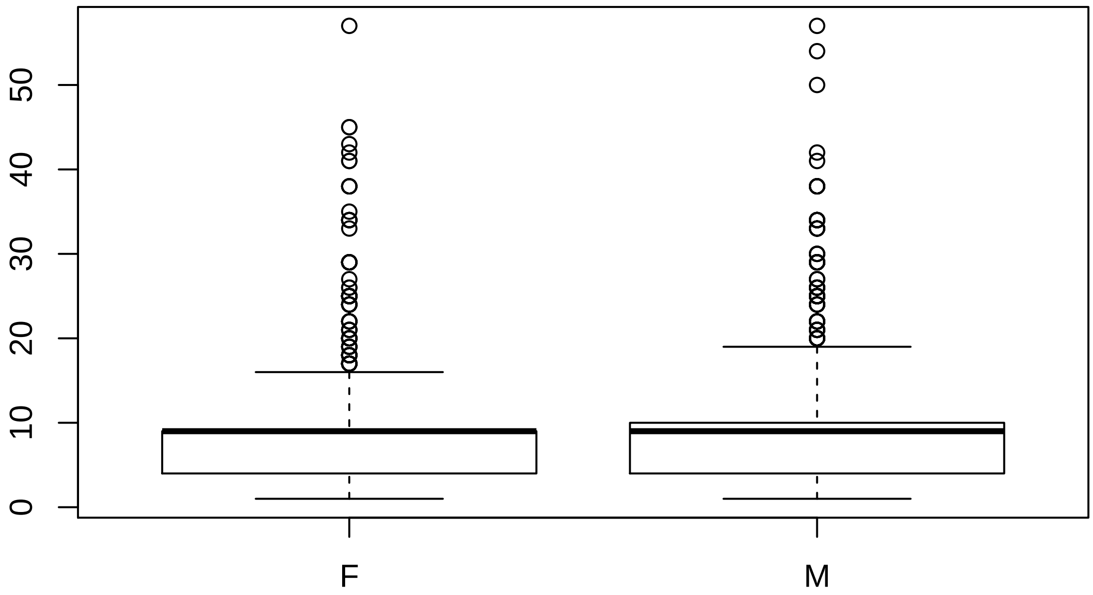


```r
boxplot(iss~ICED_23, data=trauma,  xlab = "ICED", ylab = "ISS score")
```

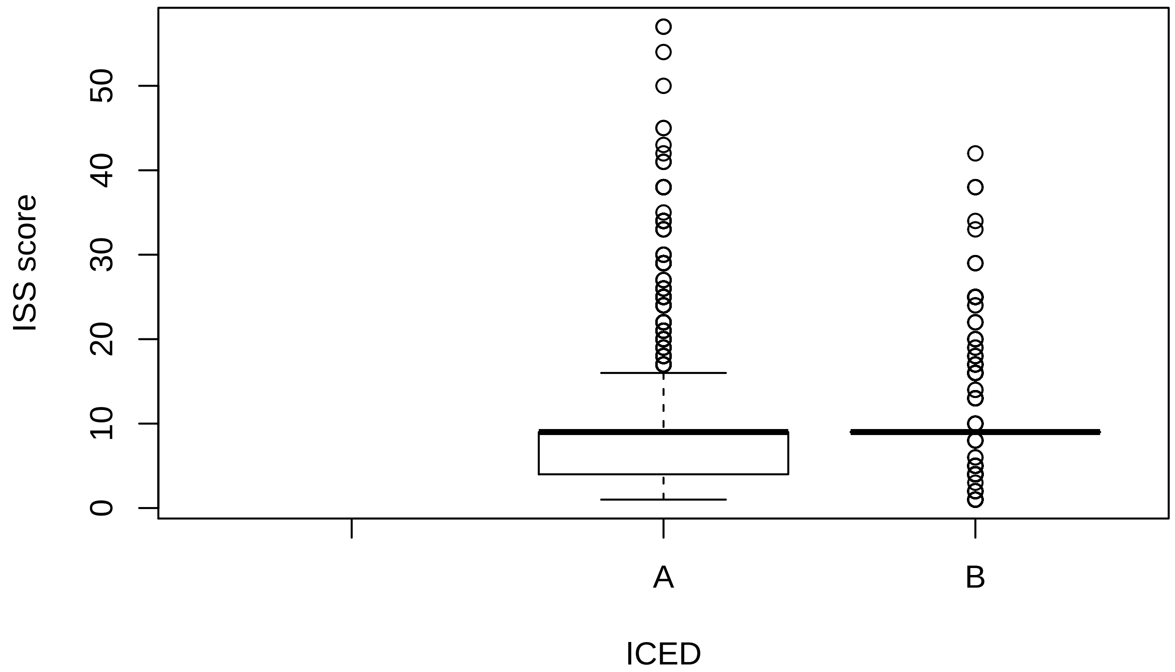


```r
CrossTable(trauma$ISS_3, trauma$Sex,
           chisq = T, fisher=F)
```

```
##
##
```

```
##    Cell Contents
## |-------------------------|
## |                       N |
## | Chi-square contribution |
## |           N / Row Total |
## |           N / Col Total |
## |         N / Table Total |
## |-------------------------|
##
##
## Total Observations in Table:  4997
##
##
##              | trauma$Sex
## trauma$ISS_3 |         F |         M | Row Total |
## -------------|-----------|-----------|-----------|
##            1 |      1974 |      2257 |      4231 |
##              |     3.445 |     2.790 |           |
##              |     0.467 |     0.533 |     0.847 |
##              |     0.883 |     0.817 |           |
##              |     0.395 |     0.452 |           |
## -------------|-----------|-----------|-----------|
##            2 |       178 |       333 |       511 |
##              |    11.222 |     9.088 |           |
##              |     0.348 |     0.652 |     0.102 |
##              |     0.080 |     0.121 |           |
##              |     0.036 |     0.067 |           |
## -------------|-----------|-----------|-----------|
##            3 |        84 |       171 |       255 |
##              |     7.943 |     6.432 |           |
##              |     0.329 |     0.671 |     0.051 |
##              |     0.038 |     0.062 |           |
##              |     0.017 |     0.034 |           |
## -------------|-----------|-----------|-----------|
## Column Total |      2236 |      2761 |      4997 |
##              |     0.447 |     0.553 |           |
## -------------|-----------|-----------|-----------|
##
##
## Statistics for All Table Factors
##
##
## Pearson's Chi-squared test
## ------------------------------------------------------------
## Chi^2 =  40.9207     d.f. =  2     p =  1.300717e-09
##
##
##
```

```
chisq.test(trauma$ISS_3, trauma$Sex)
```

```
##
##  Pearson's Chi-squared test
##
## data:  trauma$ISS_3 and trauma$Sex
```

```
## X-squared = 40.921, df = 2, p-value = 1.301e-09
```

```
CrossTable(trauma$ISS_3, trauma$ICED_23, chisq = T, fisher=F, prop.r=F, prop.c=TRUE, prop.t=F, prop.chi
```

```
##
##
##    Cell Contents
## |-------------------------|
## |                       N |
## |           N / Col Total |
## |-------------------------|
##
##
## Total Observations in Table:  4997
##
##
##              | trauma$ICED_23
## trauma$ISS_3 |         A |         B | Row Total |
## -------------|-----------|-----------|-----------|
##            1 |      3534 |       697 |      4231 |
##              |     0.851 |     0.826 |           |
## -------------|-----------|-----------|-----------|
##            2 |       417 |        94 |       511 |
##              |     0.100 |     0.111 |           |
## -------------|-----------|-----------|-----------|
##            3 |       202 |        53 |       255 |
##              |     0.049 |     0.063 |           |
## -------------|-----------|-----------|-----------|
## Column Total |      4153 |       844 |      4997 |
##              |     0.831 |     0.169 |           |
## -------------|-----------|-----------|-----------|
##
##
## Statistics for All Table Factors
##
##
## Pearson's Chi-squared test
## ------------------------------------------------------------
## Chi^2 =  4.102326     d.f. =  2     p =  0.1285853
##
##
##
```

```
##Check normality
shapiro.test(trauma$age)
```

```
##
##  Shapiro-Wilk normality test
##
## data:  trauma$age
## W = 0.97132, p-value < 2.2e-16
```

```
shapiro.test(trauma$gcs)
```

```
##
##  Shapiro-Wilk normality test
```

```
##
## data:  trauma$gcs
## W = 0.31193, p-value < 2.2e-16
```

```r
shapiro.test(trauma$pr)
```

```
##
##  Shapiro-Wilk normality test
##
## data:  trauma$pr
## W = 0.94836, p-value < 2.2e-16
```

```r
shapiro.test(trauma$sbp)
```

```
##
##  Shapiro-Wilk normality test
##
## data:  trauma$sbp
## W = 0.98901, p-value < 2.2e-16
```

```r
shapiro.test(trauma$rr)
```

```
##
##  Shapiro-Wilk normality test
##
## data:  trauma$rr
## W = 0.79387, p-value < 2.2e-16
```

```r
shapiro.test(trauma$iss)
```

```
##
##  Shapiro-Wilk normality test
##
## data:  trauma$iss
## W = 0.77652, p-value < 2.2e-16
```

```r
shapiro.test(trauma$Co_no)
```

```
##
##  Shapiro-Wilk normality test
##
## data:  trauma$Co_no
## W = 0.77698, p-value < 2.2e-16
```

```r
shapiro.test(trauma$ADday)
```

```
##
##  Shapiro-Wilk normality test
##
## data:  trauma$ADday
## W = 0.67646, p-value < 2.2e-16
```

```r
cor(trauma$age, trauma$iss)
```

```
## [1] 0.08485337
```

```r
cor.test(trauma$age, trauma$iss)
```

```
##
##  Pearson's product-moment correlation
```

```
##
## data:  trauma$age and trauma$iss
## t = 6.0187, df = 4995, p-value = 1.883e-09
## alternative hypothesis: true correlation is not equal to 0
## 95 percent confidence interval:
##  0.05726048 0.11231673
## sample estimates:
##        cor
## 0.08485337
```

Prepare counting process dataset for competing risk model

```
tmat <- trans.comprisk(2,c("Admission","Death","Discharge"))
tmat
```

```
##            to
## from         Admission Death Discharge
##   Admission        NA     1         2
##   Death            NA    NA        NA
##   Discharge        NA    NA        NA
```

```
trauma$Death=ifelse(trauma$status==1, 1, 0)
#table(trauma$Death)
trauma$Discharge=ifelse(trauma$status==2, 1, 0)
#table(trauma$Discharge)
#table(trauma$status)
#dput(names(trauma))
covs=c("Sex", "age", "alcohol", "gcs", "GCS",
"pr", "sbp", "rr",  "iss", "ISS_3","ICU", "ADday", "Co_no", "ICED_N", "ICED_23", "Co.no",
"status", "Death", "Discharge")

msbmt <- msprep(time=c(NA,"ADday","ADday"),
status=c(NA,"Death","Discharge"), data=trauma, trans=tmat,
keep=covs)
#head(msbmt)
##Transition matrix
events(msbmt)
```

```
## $Frequencies
##            to
## from         Admission Death Discharge no event total entering
##   Admission         0   114      4810       73            4997
##   Death             0     0         0      114             114
##   Discharge         0     0         0     4810            4810
##
## $Proportions
##            to
## from          Admission      Death  Discharge   no event
##   Admission 0.00000000 0.02281369 0.96257755 0.01460877
##   Death     0.00000000 0.00000000 0.00000000 1.00000000
##   Discharge 0.00000000 0.00000000 0.00000000 1.00000000
```

```
msbmt$ICED=ifelse(msbmt$ICED_23=="B", 1, 0)
#table(msbmt$ICED)
```

```
##Expand covariate
COV <-c( "Sex", "age", "alcohol", "GCS","gcs",   "sbp",
 "ISS_3","iss", "ICU", "Co_no","Co.no", "ICED")

sdata<- expand.covs(msbmt,COV,append=TRUE,longnames=FALSE)
#head(sdata)
#dput(names(sdata))
```

Nonparametric estimate of cumulative hazard function

```
#table(sdata$status)
c0 <- coxph(Surv(Tstart,Tstop,status)~strata(trans), data=sdata, method="breslow")
#msfit with Greenwood variance estimator
msf0 <- msfit(object=c0, vartype="greenwood", trans=tmat)
#plot(msf0, las=1, lty=1:2, xlab="Days after Admission" lwd=2, xlim=c(0,80))

##Estimate of transition parobability without covariates
pt0 <- probtrans(msf0, predt=0, method="greenwood")

#statecols <- heat_hcl(8, c = c(80, 30), l = c(30, 90), power = c(1/5, 2))[c(6,5,3,4,2,1)]
#ord <- c(1,2,3)
#plot(pt0, ord=c(1,2,3), xlab="Days since Admission", las=1, type="filled", col=statecols[ord])
```

```
##ICED_23==A
sdata.A=subset(sdata, sdata$ICED==0)
cA <- coxph(Surv(Tstart,Tstop,status)~strata(trans), data=sdata.A, method="breslow")

##ICED_23==B
sdata.B=subset(sdata, sdata$ICED==1)
cB <- coxph(Surv(Tstart,Tstop,status)~strata(trans), data=sdata.B, method="breslow")
#msfit with Greenwood variance estimator
#par(mfrow=c(1,2))
msfA <- msfit(object=cA, vartype="greenwood", trans=tmat)
#plot(msfA, las=1, lty=1:2, xlab="Days after Admission",lwd=2, xlim=c(0,80),ylim=c(0,7), main="ICED_23=
msfB <- msfit(object=cB, vartype="greenwood", trans=tmat)
#plot(msfB, las=1, lty=1:2, xlab="Days after Admission", lwd=2, xlim=c(0,80),ylim=c(0,7),main="ICED_23=

##Estimate of transition parobability without covariates
ptA <- probtrans(msfA, predt=0, method="greenwood")

ptB <- probtrans(msfB, predt=0, method="greenwood")
```

Create Figure 1

```
msf.A=msfA[[1]]
msf.B=msfB[[1]]

par(mfrow=c(1,2))
plot(msf.A$time[msf.A$trans==1], msf.A$Haz[msf.A$trans==1],type="s", ylim=c(0,1.0), xlim=c(0,80), ylab=
lines(msf.B$time[msf.B$trans==1], msf.B$Haz[msf.B$trans==1],
```

```
     type="s", ylim=c(0,1.0), xlim=c(0,80), col="blue", main="Transition to death",cex=1,
     lwd=2)
legend("topleft", col=c("blue", "orange"), lty=c(1,1),lwd = c(2,2),
 legend=c("ICED 2/3", "ICED 0/1"),cex=1 )

plot(msf.A$time[msf.A$trans==2], msf.A$Haz[msf.A$trans==2],
     type="s", ylim=c(0,7.0), xlim=c(0,80), ylab="Cumulative hazards", xlab="Days since admission", col=
lines(msf.B$time[msf.B$trans==2], msf.B$Haz[msf.B$trans==2],
     type="s", ylim=c(0,1.0), xlim=c(0,80), col="blue", main="Transplant to Death",cex=1, lwd=2)
legend("topleft", col=c("blue", "orange"), lty=c(1,1),lwd = c(2,2),
 legend=c("ICED 2/3", "ICED 0/1"),cex=1 )
```

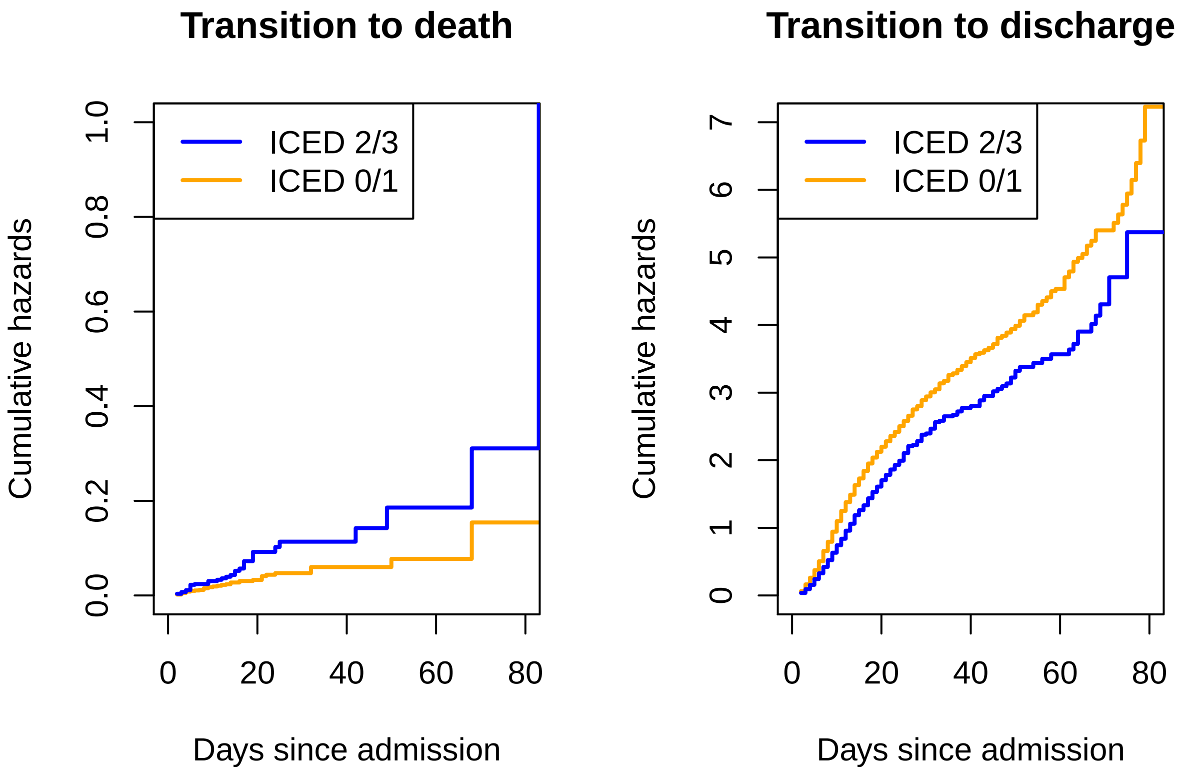

Create Table 2

```
#summary(ptA, from = 1)
library(reshape)

tmp <- tail(subset(ptA[[1]],time<=20),1)
tmp
```

```
##    time    pstate1    pstate2   pstate3         se1         se2
## 20   20 0.09214582 0.01578171 0.8920725 0.004526698 0.001942036
##             se3
## 20 0.004853112
```

```
tmp.prob <- c(tmp$pstate1,tmp$pstate2,tmp$pstate3)

Prob.occ1.tx <- data.frame(P=tmp.prob,
```

```
 lci=tmp.prob-qnorm(0.975)*c(tmp$se1,tmp$se2,tmp$se3),
 uci=tmp.prob+qnorm(0.975)*c(tmp$se1,tmp$se2,tmp$se3))
#Prob.occ1.tx
Prob.occ1.tx=round(Prob.occ1.tx*100, digits=1)

State=c("pstate1", "pstate2", "pstate3")

Prob.occ1.tx=rename(Prob.occ1.tx, c(P="20-day",
        lci="Lower CI", uci="Upper CI"))
Prob.occ1.tx=cbind(State, Prob.occ1.tx)
#Prob.occ1.tx

tmp <- tail(subset(ptA[[1]],time<=40),1)
tmp
```

```
##    time   pstate1    pstate2   pstate3         se1         se2        se3
## 40   40 0.0229304 0.01752172 0.9595479 0.002362883 0.002046903 0.00309395
```

```
tmp.prob <- c(tmp$pstate1,tmp$pstate2,tmp$pstate3)

Prob.occ2.tx <- data.frame(P=tmp.prob,
 lci=tmp.prob-qnorm(0.975)*c(tmp$se1,tmp$se2,tmp$se3),
 uci=tmp.prob+qnorm(0.975)*c(tmp$se1,tmp$se2,tmp$se3))
#Prob.occ2.tx
Prob.occ2.tx=round(Prob.occ2.tx*100, digits=1)

Prob.occ2.tx=rename(Prob.occ2.tx, c(P="40-day",
        lci="Lower CI", uci="Upper CI"))
#Prob.occ2.tx

tmp <- tail(subset(ptA[[1]],time<=60),1)
tmp
```

```
##    time     pstate1    pstate2   pstate3         se1         se2
## 58   59 0.007823647 0.01777685 0.9743995 0.001408079 0.002062193
##             se3
## 58 0.002482906
```

```
tmp.prob <- c(tmp$pstate1,tmp$pstate2,tmp$pstate3)

Prob.occ3.tx <- data.frame(P=tmp.prob,
 lci=tmp.prob-qnorm(0.975)*c(tmp$se1,tmp$se2,tmp$se3),
 uci=tmp.prob+qnorm(0.975)*c(tmp$se1,tmp$se2,tmp$se3))
#Prob.occ3.tx
Prob.occ3.tx=round(Prob.occ3.tx*100, digits=1)

Prob.occ3.tx=rename(Prob.occ3.tx, c(P="60-day",
        lci="Lower CI", uci="Upper CI"))
#Prob.occ3.tx

Prob.tx=cbind(Prob.occ1.tx,Prob.occ2.tx,Prob.occ3.tx)

#Prob.tx

##ICED 2/3
```

```r
tmp <- tail(subset(ptB[[1]],time<=20),1)
tmp
```

```
##    time  pstate1   pstate2   pstate3        se1         se2        se3
## 20   20 0.1507595 0.0405589 0.8086816 0.01238922 0.006813516 0.01361021
```

```r
tmp.prob <- c(tmp$pstate1,tmp$pstate2,tmp$pstate3)

Prob.occ1.ntx <- data.frame(P=tmp.prob,
 lci=tmp.prob-qnorm(0.975)*c(tmp$se1,tmp$se2,tmp$se3),
 uci=tmp.prob+qnorm(0.975)*c(tmp$se1,tmp$se2,tmp$se3))
#Prob.occ1.ntx

Prob.occ1.ntx=round(Prob.occ1.ntx*100, digits=1)

Prob.occ1.ntx=rename(Prob.occ1.ntx, c(P="20-day",
        lci="Lower CI", uci="Upper CI"))
Prob.occ1.ntx=cbind(State, Prob.occ1.ntx)
#Prob.occ1.ntx

tmp <- tail(subset(ptB[[1]],time<=40),1)
tmp
```

```
##    time    pstate1    pstate2  pstate3         se1         se2        se3
## 38   40 0.04706856 0.04303946 0.909892 0.007482966 0.007017819 0.01001547
```

```r
tmp.prob <- c(tmp$pstate1,tmp$pstate2,tmp$pstate3)

Prob.occ2.ntx <- data.frame(P=tmp.prob,
 lci=tmp.prob-qnorm(0.975)*c(tmp$se1,tmp$se2,tmp$se3),
 uci=tmp.prob+qnorm(0.975)*c(tmp$se1,tmp$se2,tmp$se3))
#Prob.occ2.ntx

Prob.occ2.ntx=round(Prob.occ2.ntx*100, digits=1)

Prob.occ2.ntx=rename(Prob.occ2.ntx, c(P="40-day",
        lci="Lower CI", uci="Upper CI"))
#Prob.occ2.ntx

tmp <- tail(subset(ptB[[1]],time<=60),1)
tmp
```

```
##    time   pstate1    pstate2  pstate3         se1       se2         se3
## 50   58 0.0196119 0.04578512 0.934603 0.005056112 0.0072598 0.008704372
```

```r
tmp.prob <- c(tmp$pstate1,tmp$pstate2,tmp$pstate3)

Prob.occ3.ntx <- data.frame(P=tmp.prob,
 lci=tmp.prob-qnorm(0.975)*c(tmp$se1,tmp$se2,tmp$se3),
 uci=tmp.prob+qnorm(0.975)*c(tmp$se1,tmp$se2,tmp$se3))
#Prob.occ3.ntx
Prob.occ3.ntx=round(Prob.occ3.ntx*100, digits=1)

Prob.occ3.ntx=rename(Prob.occ3.ntx, c(P="60-day",
        lci="Lower CI", uci="Upper CI"))
```

```r
#Prob.occ3.ntx

Prob.ntx=cbind(Prob.occ1.ntx,Prob.occ2.ntx,Prob.occ3.ntx)

#Prob.ntx

#FlexTable(Prob.ntx)

Prob=rbind(Prob.tx, Prob.ntx)

Type=c(rep("ICED 0/1",3), rep("ICED 2/3",3))
Prob=cbind(Type, Prob)
Prob
```

```
##       Type   State 20-day Lower CI Upper CI 40-day Lower CI Upper CI
## 1 ICED 0/1 pstate1    9.2      8.3     10.1    2.3      1.8      2.8
## 2 ICED 0/1 pstate2    1.6      1.2      2.0    1.8      1.4      2.2
## 3 ICED 0/1 pstate3   89.2     88.3     90.2   96.0     95.3     96.6
## 4 ICED 2/3 pstate1   15.1     12.6     17.5    4.7      3.2      6.2
## 5 ICED 2/3 pstate2    4.1      2.7      5.4    4.3      2.9      5.7
## 6 ICED 2/3 pstate3   80.9     78.2     83.5   91.0     89.0     93.0
##   60-day Lower CI Upper CI
## 1    0.8      0.5      1.1
## 2    1.8      1.4      2.2
## 3   97.4     97.0     97.9
## 4    2.0      1.0      3.0
## 5    4.6      3.2      6.0
## 6   93.5     91.8     95.2
```

Cause-specific Cox model

```r
c0 <- coxph(Surv(Tstart,Tstop,status) ~
Sex.1+age.1+alcohol.1+gcs.1+sbp.1+iss.1+ICU.1+
Co_no.1+ICED.1+
Sex.2+age.1+alcohol.2+gcs.1+sbp.2+iss.2+ICU.2+
Co_no.2+ICED.2
+ strata(trans),
data=sdata, method="breslow")

summary(c0)
```

```
## Call:
## coxph(formula = Surv(Tstart, Tstop, status) ~ Sex.1 + age.1 +
##     alcohol.1 + gcs.1 + sbp.1 + iss.1 + ICU.1 + Co_no.1 + ICED.1 +
##     Sex.2 + age.1 + alcohol.2 + gcs.1 + sbp.2 + iss.2 + ICU.2 +
##     Co_no.2 + ICED.2 + strata(trans), data = sdata, method = "breslow")
##
##   n= 9994, number of events= 4924
##
##                 coef  exp(coef)   se(coef)      z Pr(>|z|)
## Sex.1      2.762e-01  1.318e+00  2.029e-01  1.361  0.17358
## age.1      4.002e-02  1.041e+00  7.071e-03  5.659 1.52e-08 ***
## alcohol.1  2.806e-01  1.324e+00  2.835e-01  0.990  0.32238
```

```
## gcs.1     -1.803e-01  8.350e-01  2.506e-02  -7.197 6.17e-13 ***
## sbp.1     -3.073e-05  1.000e+00  2.379e-03  -0.013  0.98969
## iss.1      5.711e-02  1.059e+00  1.067e-02   5.351 8.73e-08 ***
## ICU.1      1.479e+00  4.389e+00  2.951e-01   5.012 5.38e-07 ***
## Co_no.1   -7.675e-02  9.261e-01  1.000e-01  -0.767  0.44291
## ICED.1     6.363e-01  1.889e+00  2.387e-01   2.666  0.00768 **
## Sex.2     -8.013e-02  9.230e-01  2.980e-02  -2.689  0.00717 **
## alcohol.2  2.581e-02  1.026e+00  6.356e-02   0.406  0.68472
## sbp.2     -3.209e-04  9.997e-01  4.934e-04  -0.650  0.51547
## iss.2     -3.641e-02  9.642e-01  3.235e-03 -11.258  < 2e-16 ***
## ICU.2     -6.485e-01  5.228e-01  5.514e-02 -11.761  < 2e-16 ***
## Co_no.2   -7.047e-02  9.320e-01  1.567e-02  -4.497 6.90e-06 ***
## ICED.2    -2.380e-01  7.882e-01  4.432e-02  -5.369 7.90e-08 ***
## ---
## Signif. codes:  0 '***' 0.001 '**' 0.01 '*' 0.05 '.' 0.1 ' ' 1
##
##           exp(coef) exp(-coef) lower .95 upper .95
## Sex.1        1.3180     0.7587    0.8855    1.9619
## age.1        1.0408     0.9608    1.0265    1.0554
## alcohol.1    1.3239     0.7553    0.7595    2.3078
## gcs.1        0.8350     1.1976    0.7950    0.8770
## sbp.1        1.0000     1.0000    0.9953    1.0046
## iss.1        1.0588     0.9445    1.0369    1.0811
## ICU.1        4.3887     0.2279    2.4613    7.8253
## Co_no.1      0.9261     1.0798    0.7613    1.1267
## ICED.1       1.8894     0.5293    1.1834    3.0165
## Sex.2        0.9230     1.0834    0.8706    0.9785
## alcohol.2    1.0261     0.9745    0.9060    1.1623
## sbp.2        0.9997     1.0003    0.9987    1.0006
## iss.2        0.9642     1.0371    0.9581    0.9704
## ICU.2        0.5228     1.9127    0.4693    0.5825
## Co_no.2      0.9320     1.0730    0.9038    0.9610
## ICED.2       0.7882     1.2687    0.7226    0.8598
##
## Concordance= 0.646  (se = 0.006 )
## Rsquare= 0.107   (max possible= 0.999 )
## Likelihood ratio test= 1130  on 16 df,   p=0
## Wald test            = 946  on 16 df,   p=0
## Score (logrank) test = 1166  on 16 df,   p=0
```

```
library(MASS)
##Model selction using AIC
summary(stepAIC(c0, direction="both"))
```

```
## Start:  AIC=73551.73
## Surv(Tstart, Tstop, status) ~ Sex.1 + age.1 + alcohol.1 + gcs.1 +
##     sbp.1 + iss.1 + ICU.1 + Co_no.1 + ICED.1 + Sex.2 + age.1 +
##     alcohol.2 + gcs.1 + sbp.2 + iss.2 + ICU.2 + Co_no.2 + ICED.2 +
##     strata(trans)
##
##             Df   AIC
## - sbp.1      1 73550
## - alcohol.2  1 73550
## - sbp.2      1 73550
## - Co_no.1    1 73550
```

```
## - alcohol.1  1 73551
## - Sex.1      1 73552
## <none>         73552
## - ICED.1     1 73557
## - Sex.2      1 73557
## - Co_no.2    1 73571
## - ICU.1      1 73576
## - iss.1      1 73577
## - ICED.2     1 73579
## - age.1      1 73584
## - gcs.1      1 73601
## - iss.2      1 73686
## - ICU.2      1 73702
##
## Step:  AIC=73549.73
## Surv(Tstart, Tstop, status) ~ Sex.1 + age.1 + alcohol.1 + gcs.1 +
##     iss.1 + ICU.1 + Co_no.1 + ICED.1 + Sex.2 + alcohol.2 + sbp.2 +
##     iss.2 + ICU.2 + Co_no.2 + ICED.2 + strata(trans)
##
##             Df   AIC
## - alcohol.2  1 73548
## - sbp.2      1 73548
## - Co_no.1    1 73548
## - alcohol.1  1 73549
## - Sex.1      1 73550
## <none>         73550
## + sbp.1      1 73552
## - ICED.1     1 73555
## - Sex.2      1 73555
## - Co_no.2    1 73569
## - ICU.1      1 73574
## - iss.1      1 73575
## - ICED.2     1 73577
## - age.1      1 73583
## - gcs.1      1 73599
## - iss.2      1 73684
## - ICU.2      1 73700
##
## Step:  AIC=73547.9
## Surv(Tstart, Tstop, status) ~ Sex.1 + age.1 + alcohol.1 + gcs.1 +
##     iss.1 + ICU.1 + Co_no.1 + ICED.1 + Sex.2 + sbp.2 + iss.2 +
##     ICU.2 + Co_no.2 + ICED.2 + strata(trans)
##
##             Df   AIC
## - sbp.2      1 73546
## - Co_no.1    1 73547
## - alcohol.1  1 73547
## - Sex.1      1 73548
## <none>         73548
## + alcohol.2  1 73550
## + sbp.1      1 73550
## - ICED.1     1 73553
## - Sex.2      1 73553
## - Co_no.2    1 73567
```

```
## - ICU.1      1 73573
## - iss.1      1 73573
## - ICED.2     1 73576
## - age.1      1 73581
## - gcs.1      1 73597
## - iss.2      1 73683
## - ICU.2      1 73699
##
## Step:  AIC=73546.37
## Surv(Tstart, Tstop, status) ~ Sex.1 + age.1 + alcohol.1 + gcs.1 +
##     iss.1 + ICU.1 + Co_no.1 + ICED.1 + Sex.2 + iss.2 + ICU.2 +
##     Co_no.2 + ICED.2 + strata(trans)
##
##             Df   AIC
## - Co_no.1    1 73545
## - alcohol.1  1 73545
## - Sex.1      1 73546
## <none>         73546
## + sbp.2      1 73548
## + alcohol.2  1 73548
## + sbp.1      1 73548
## - Sex.2      1 73551
## - ICED.1     1 73551
## - Co_no.2    1 73567
## - ICU.1      1 73571
## - iss.1      1 73572
## - ICED.2     1 73574
## - age.1      1 73580
## - gcs.1      1 73595
## - iss.2      1 73681
## - ICU.2      1 73697
##
## Step:  AIC=73544.98
## Surv(Tstart, Tstop, status) ~ Sex.1 + age.1 + alcohol.1 + gcs.1 +
##     iss.1 + ICU.1 + ICED.1 + Sex.2 + iss.2 + ICU.2 + Co_no.2 +
##     ICED.2 + strata(trans)
##
##             Df   AIC
## - alcohol.1  1 73544
## - Sex.1      1 73545
## <none>         73545
## + Co_no.1    1 73546
## + sbp.2      1 73547
## + alcohol.2  1 73547
## + sbp.1      1 73547
## - ICED.1     1 73549
## - Sex.2      1 73550
## - Co_no.2    1 73565
## - iss.1      1 73570
## - ICU.1      1 73570
## - ICED.2     1 73573
## - age.1      1 73579
## - gcs.1      1 73593
## - iss.2      1 73679
```

```
## - ICU.2      1 73695
##
## Step:  AIC=73544.04
## Surv(Tstart, Tstop, status) ~ Sex.1 + age.1 + gcs.1 + iss.1 +
##     ICU.1 + ICED.1 + Sex.2 + iss.2 + ICU.2 + Co_no.2 + ICED.2 +
##     strata(trans)
##
##             Df   AIC
## <none>         73544
## - Sex.1      1 73544
## + alcohol.1  1 73545
## + Co_no.1    1 73545
## + sbp.2      1 73546
## + alcohol.2  1 73546
## + sbp.1      1 73546
## - ICED.1     1 73548
## - Sex.2      1 73549
## - Co_no.2    1 73564
## - ICU.1      1 73569
## - iss.1      1 73570
## - ICED.2     1 73572
## - age.1      1 73578
## - gcs.1      1 73597
## - iss.2      1 73678
## - ICU.2      1 73694
```

```
## Call:
## coxph(formula = Surv(Tstart, Tstop, status) ~ Sex.1 + age.1 +
##     gcs.1 + iss.1 + ICU.1 + ICED.1 + Sex.2 + iss.2 + ICU.2 +
##     Co_no.2 + ICED.2 + strata(trans), data = sdata, method = "breslow")
##
##   n= 9994, number of events= 4924
##
##              coef exp(coef)  se(coef)       z Pr(>|z|)
## Sex.1    0.292580  1.339880  0.199728   1.465  0.14295
## age.1    0.036283  1.036950  0.006339   5.724 1.04e-08 ***
## gcs.1   -0.182597  0.833103  0.024661  -7.404 1.32e-13 ***
## iss.1    0.057096  1.058757  0.010368   5.507 3.65e-08 ***
## ICU.1    1.482604  4.404401  0.295370   5.019 5.18e-07 ***
## ICED.1   0.559026  1.748968  0.218637   2.557  0.01056 *
## Sex.2   -0.076984  0.925905  0.029452  -2.614  0.00895 **
## iss.2   -0.036240  0.964409  0.003224 -11.242  < 2e-16 ***
## ICU.2   -0.644047  0.525163  0.054701 -11.774  < 2e-16 ***
## Co_no.2 -0.072312  0.930241  0.015495  -4.667 3.06e-06 ***
## ICED.2  -0.238057  0.788158  0.044288  -5.375 7.65e-08 ***
## ---
## Signif. codes:  0 '***' 0.001 '**' 0.01 '*' 0.05 '.' 0.1 ' ' 1
##
##         exp(coef) exp(-coef) lower .95 upper .95
## Sex.1      1.3399     0.7463    0.9059    1.9819
## age.1      1.0369     0.9644    1.0241    1.0499
## gcs.1      0.8331     1.2003    0.7938    0.8744
## iss.1      1.0588     0.9445    1.0375    1.0805
## ICU.1      4.4044     0.2270    2.4687    7.8579
```

```
## ICED.1     1.7490     0.5718    1.1394    2.6846
## Sex.2      0.9259     1.0800    0.8740    0.9809
## iss.2      0.9644     1.0369    0.9583    0.9705
## ICU.2      0.5252     1.9042    0.4718    0.5846
## Co_no.2    0.9302     1.0750    0.9024    0.9589
## ICED.2     0.7882     1.2688    0.7226    0.8596
##
## Concordance= 0.646  (se = 0.006 )
## Rsquare= 0.107   (max possible= 0.999 )
## Likelihood ratio test= 1128  on 11 df,   p=0
## Wald test            = 948.3  on 11 df,   p=0
## Score (logrank) test = 1163  on 11 df,   p=0
```

```
#########################################
c3 <- coxph(formula = Surv(Tstart, Tstop, status) ~ Sex.1 + age.1 +
    gcs.1 + iss.1 + ICU.1 + ICED.1 + Sex.2 + iss.2 + ICU.2 +
    Co_no.2 + ICED.2 + strata(trans), data = sdata, method = "breslow")

summary(c3)
```

```
## Call:
## coxph(formula = Surv(Tstart, Tstop, status) ~ Sex.1 + age.1 +
##     gcs.1 + iss.1 + ICU.1 + ICED.1 + Sex.2 + iss.2 + ICU.2 +
##     Co_no.2 + ICED.2 + strata(trans), data = sdata, method = "breslow")
##
##   n= 9994, number of events= 4924
##
##              coef exp(coef)  se(coef)       z Pr(>|z|)
## Sex.1    0.292580  1.339880  0.199728   1.465  0.14295
## age.1    0.036283  1.036950  0.006339   5.724 1.04e-08 ***
## gcs.1   -0.182597  0.833103  0.024661  -7.404 1.32e-13 ***
## iss.1    0.057096  1.058757  0.010368   5.507 3.65e-08 ***
## ICU.1    1.482604  4.404401  0.295370   5.019 5.18e-07 ***
## ICED.1   0.559026  1.748968  0.218637   2.557  0.01056 *
## Sex.2   -0.076984  0.925905  0.029452  -2.614  0.00895 **
## iss.2   -0.036240  0.964409  0.003224 -11.242  < 2e-16 ***
## ICU.2   -0.644047  0.525163  0.054701 -11.774  < 2e-16 ***
## Co_no.2 -0.072312  0.930241  0.015495  -4.667 3.06e-06 ***
## ICED.2  -0.238057  0.788158  0.044288  -5.375 7.65e-08 ***
## ---
## Signif. codes:  0 '***' 0.001 '**' 0.01 '*' 0.05 '.' 0.1 ' ' 1
##
##         exp(coef) exp(-coef) lower .95 upper .95
## Sex.1      1.3399     0.7463    0.9059    1.9819
## age.1      1.0369     0.9644    1.0241    1.0499
## gcs.1      0.8331     1.2003    0.7938    0.8744
## iss.1      1.0588     0.9445    1.0375    1.0805
## ICU.1      4.4044     0.2270    2.4687    7.8579
## ICED.1     1.7490     0.5718    1.1394    2.6846
## Sex.2      0.9259     1.0800    0.8740    0.9809
## iss.2      0.9644     1.0369    0.9583    0.9705
## ICU.2      0.5252     1.9042    0.4718    0.5846
## Co_no.2    0.9302     1.0750    0.9024    0.9589
## ICED.2     0.7882     1.2688    0.7226    0.8596
##
```

```
## Concordance= 0.646  (se = 0.006 )
## Rsquare= 0.107   (max possible= 0.999 )
## Likelihood ratio test= 1128  on 11 df,   p=0
## Wald test            = 948.3  on 11 df,   p=0
## Score (logrank) test = 1163  on 11 df,   p=0
```

```
##Check proportial hazard assumption of Cox model
zp.3=cox.zph(c3)
zp.3
```

```
##              rho    chisq        p
## Sex.1    0.00362   0.0666 7.96e-01
## age.1    0.01022   0.6397 4.24e-01
## gcs.1    0.01307   0.8169 3.66e-01
## iss.1   -0.05622   9.1041 2.55e-03
## ICU.1   -0.02650   3.8093 5.10e-02
## ICED.1   0.01159   0.6517 4.20e-01
## Sex.2   -0.04605  10.4578 1.22e-03
## iss.2    0.07559  31.0989 2.45e-08
## ICU.2    0.06653  21.5392 3.47e-06
## Co_no.2  0.02386   2.8702 9.02e-02
## ICED.2   0.00908   0.4028 5.26e-01
## GLOBAL        NA 173.1247 0.00e+00
```

```
#plot(zp.3, resid=F)
```

Time to death outcome

```
trans1=subset(sdata, trans==1)
##Cut time point of 14 days
trans.1=survSplit(Surv(Tstart,Tstop,status) ~., trans1,cut=c(14),start="tstart", episode ="timegroup")

median(trans1$iss.1)
```

```
## [1] 9
```

```
# Center ISS at its median (9)
trans1$IssMed <- trans1$iss.1 - 9

##Model Comparison
trans1$age10=trans1$age.1/10

T2 <- coxph(Surv(Tstart,Tstop,status) ~
pspline(iss.1, df=5)+pspline(sbp.1, df=2) +Sex.1+ age10 + gcs.1 +  ICU.1 + ICED.1
,data=trans1, method="breslow")

summary(T2)
```

```
## Call:
## coxph(formula = Surv(Tstart, Tstop, status) ~ pspline(iss.1,
##     df = 5) + pspline(sbp.1, df = 2) + Sex.1 + age10 + gcs.1 +
##     ICU.1 + ICED.1, data = trans1, method = "breslow")
##
##   n= 4997, number of events= 114
##
```

```
##                           coef      se(coef) se2      Chisq DF p
## pspline(iss.1, df = 5), l  0.068179 0.012355 0.012316 30.45 1  3.4e-08
## pspline(iss.1, df = 5), n                             12.75 4  1.3e-02
## pspline(sbp.1, df = 2), l -0.002039 0.002226 0.002217  0.84 1  3.6e-01
## pspline(sbp.1, df = 2), n                              3.18 1  7.5e-02
## Sex.1                      0.302098 0.200573 0.200165  2.27 1  1.3e-01
## age10                      0.342329 0.063619 0.063501 28.95 1  7.4e-08
## gcs.1                     -0.169499 0.025210 0.024919 45.20 1  1.8e-11
## ICU.1                      1.088453 0.341122 0.337428 10.18 1  1.4e-03
## ICED.1                     0.469040 0.220384 0.219301  4.53 1  3.3e-02
##
##              exp(coef) exp(-coef) lower .95 upper .95
## ps(iss.1)3      0.9077    1.10166  0.127577    6.4585
## ps(iss.1)4      0.9809    1.01952  0.057544   16.7191
## ps(iss.1)5      1.5808    0.63258  0.082759   30.1965
## ps(iss.1)6      2.3140    0.43215  0.127988   41.8371
## ps(iss.1)7      3.0863    0.32402  0.174364   54.6271
## ps(iss.1)8      6.9461    0.14397  0.399827  120.6715
## ps(iss.1)9      9.9679    0.10032  0.557874  178.1042
## ps(iss.1)10     9.3870    0.10653  0.520009  169.4504
## ps(iss.1)11    11.1243    0.08989  0.605808  204.2712
## ps(iss.1)12    16.3481    0.06117  0.797842  334.9806
## ps(iss.1)13    14.4610    0.06915  0.614864  340.1091
## ps(iss.1)14     5.6757    0.17619  0.215428  149.5339
## ps(iss.1)15     1.5716    0.63629  0.035904   68.7920
## ps(iss.1)16     0.4281    2.33617  0.002527   72.5102
## ps(sbp.1)3      0.5727    1.74610  0.207976    1.5771
## ps(sbp.1)4      0.3372    2.96598  0.070646    1.6091
## ps(sbp.1)5      0.2318    4.31378  0.039522    1.3597
## ps(sbp.1)6      0.2003    4.99318  0.033379    1.2017
## ps(sbp.1)7      0.2309    4.33165  0.038424    1.3870
## ps(sbp.1)8      0.2841    3.51952  0.042893    1.8821
## ps(sbp.1)9      0.3501    2.85655  0.034935    3.5080
## Sex.1           1.3527    0.73927  0.913001    2.0041
## age10           1.4082    0.71011  1.243138    1.5952
## gcs.1           0.8441    1.18471  0.803394    0.8868
## ICU.1           2.9697    0.33674  1.521757    5.7953
## ICED.1          1.5985    0.62560  1.037793    2.4620
##
## Iterations: 5 outer, 19 Newton-Raphson
##      Theta= 0.595658
##      Theta= 0.8642118
## Degrees of freedom for terms= 5 2 1 1 1 1 1
## Concordance= 0.93  (se = 0.032 )
## Likelihood ratio test= 328.4  on 11.94 df,   p=0
```

```r
zp.T2=cox.zph(T2)
zp.T2
```

```
##                  rho    chisq      p
## ps(iss.1)3   0.04001 8.19e-02 0.7747
## ps(iss.1)4   0.04070 1.39e-01 0.7096
## ps(iss.1)5   0.03503 1.31e-01 0.7173
## ps(iss.1)6   0.01694 3.11e-02 0.8599
## ps(iss.1)7   0.01276 1.66e-02 0.8973
```

```
## ps(iss.1)8   0.01682 2.90e-02 0.8647
## ps(iss.1)9   0.00685 4.85e-03 0.9445
## ps(iss.1)10 -0.00104 1.12e-04 0.9916
## ps(iss.1)11 -0.00592 3.59e-03 0.9522
## ps(iss.1)12 -0.04427 2.08e-01 0.6483
## ps(iss.1)13 -0.10763 1.29e+00 0.2567
## ps(iss.1)14 -0.18100 3.41e+00 0.0649
## ps(iss.1)15 -0.25098 4.83e+00 0.0280
## ps(iss.1)16 -0.30692 4.17e+00 0.0411
## ps(sbp.1)3  -0.05395 1.01e-01 0.7509
## ps(sbp.1)4  -0.05271 1.51e-01 0.6972
## ps(sbp.1)5  -0.04965 1.79e-01 0.6719
## ps(sbp.1)6  -0.06096 3.06e-01 0.5799
## ps(sbp.1)7  -0.08770 6.65e-01 0.4146
## ps(sbp.1)8  -0.09931 8.64e-01 0.3526
## ps(sbp.1)9  -0.10079 7.49e-01 0.3868
## Sex.1        0.03400 1.39e-01 0.7089
## age10        0.06829 6.27e-01 0.4286
## gcs.1        0.03806 1.52e-01 0.6966
## ICU.1       -0.11341 1.51e+00 0.2191
## ICED.1       0.05655 3.59e-01 0.5489
## GLOBAL            NA 3.46e+01 0.1214
```

```
#plot(zp.T2, resid=F)

T3 <- coxph(Surv(Tstart,Tstop,status) ~
age10 + gcs.1 + pspline(iss.1, df=6) + ICU.1 + ICED.1
,data=trans1, method="breslow")

summary(T3)
```

```
## Call:
## coxph(formula = Surv(Tstart, Tstop, status) ~ age10 + gcs.1 +
##     pspline(iss.1, df = 6) + ICU.1 + ICED.1, data = trans1, method = "breslow")
##
##   n= 4997, number of events= 114
##
##                            coef     se(coef) se2     Chisq DF   p
## age10                       0.32130 0.06181  0.06171 27.02 1.00 2.0e-07
## gcs.1                      -0.17513 0.02502  0.02490 48.98 1.00 2.6e-12
## pspline(iss.1, df = 6), l  0.06797 0.01250  0.01249 29.59 1.00 5.3e-08
## pspline(iss.1, df = 6), n                            13.48 4.93 1.8e-02
## ICU.1                       1.15921 0.35109  0.34798 10.90 1.00 9.6e-04
## ICED.1                      0.47209 0.22001  0.21925  4.60 1.00 3.2e-02
##
##              exp(coef) exp(-coef) lower .95 upper .95
## age10           1.3789    0.72521 1.2215984    1.5565
## gcs.1           0.8393    1.19141 0.7991713    0.8815
## ps(iss.1)3      0.5865    1.70505 0.0779222    4.4143
## ps(iss.1)4      0.4158    2.40473 0.0212653    8.1320
## ps(iss.1)5      0.5842    1.71164 0.0266082   12.8280
## ps(iss.1)6      0.9123    1.09610 0.0425974   19.5395
## ps(iss.1)7      1.1170    0.89525 0.0543109   22.9732
## ps(iss.1)8      1.1993    0.83384 0.0570797   25.1971
## ps(iss.1)9      2.4783    0.40350 0.1209856   50.7675
```

```
## ps(iss.1)10    4.4416    0.22515 0.2151778   91.6800
## ps(iss.1)11    4.1281    0.24224 0.1940249   87.8307
## ps(iss.1)12    3.8925    0.25690 0.1822787   83.1235
## ps(iss.1)13    4.8751    0.20512 0.2235732  106.3036
## ps(iss.1)14    8.1450    0.12277 0.3464999  191.4607
## ps(iss.1)15   11.3821    0.08786 0.4208257  307.8523
## ps(iss.1)16    7.2899    0.13718 0.2357599  225.4087
## ps(iss.1)17    2.2564    0.44318 0.0642570   79.2364
## ps(iss.1)18    0.5516    1.81287 0.0084635   35.9516
## ps(iss.1)19    0.1335    7.48998 0.0004708   37.8643
## ICU.1          3.1874    0.31373 1.6017342    6.3428
## ICED.1         1.6033    0.62369 1.0417281    2.4678
##
## Iterations: 2 outer, 8 Newton-Raphson
##      Theta= 0.2957166
## Degrees of freedom for terms= 1.0 1.0 5.9 1.0 1.0
## Concordance= 0.932  (se = 0.032 )
## Likelihood ratio test= 325.6  on 9.89 df,   p=0
```

```
zp.T3=cox.zph(T3)
zp.T3
```

```
##                   rho    chisq      p
## age10         0.04277 2.41e-01 0.6232
## gcs.1         0.03164 1.15e-01 0.7342
## ps(iss.1)3    0.02922 4.88e-02 0.8253
## ps(iss.1)4    0.02952 8.00e-02 0.7774
## ps(iss.1)5    0.03154 1.21e-01 0.7284
## ps(iss.1)6    0.01849 4.35e-02 0.8348
## ps(iss.1)7    0.00303 1.15e-03 0.9730
## ps(iss.1)8    0.00322 1.24e-03 0.9720
## ps(iss.1)9    0.00783 7.48e-03 0.9311
## ps(iss.1)10   0.00236 6.84e-04 0.9791
## ps(iss.1)11  -0.01092 1.46e-02 0.9039
## ps(iss.1)12  -0.01133 1.56e-02 0.9006
## ps(iss.1)13  -0.00236 6.70e-04 0.9794
## ps(iss.1)14  -0.01595 3.07e-02 0.8608
## ps(iss.1)15  -0.06106 4.73e-01 0.4916
## ps(iss.1)16  -0.12141 1.85e+00 0.1734
## ps(iss.1)17  -0.18706 3.93e+00 0.0474
## ps(iss.1)18  -0.24500 4.78e+00 0.0288
## ps(iss.1)19  -0.28976 3.88e+00 0.0488
## ICU.1        -0.09873 1.18e+00 0.2779
## ICED.1        0.03779 1.64e-01 0.6853
## GLOBAL             NA 3.22e+01 0.0556
```

```
anova(T2, T3)
```

```
## Analysis of Deviance Table
##  Cox model: response is  Surv(Tstart, Tstop, status)
##  Model 1: ~ pspline(iss.1, df = 5) + pspline(sbp.1, df = 2) + Sex.1 + age10 + gcs.1 + ICU.1 + ICED.1
##  Model 2: ~ age10 + gcs.1 + pspline(iss.1, df = 6) + ICU.1 + ICED.1
##    loglik  Chisq     Df P(>|Chi|)
## 1 -691.29
## 2 -692.65 2.7133 2.0471    0.2658
```

```r
T.sex <- coxph(Surv(Tstart,Tstop,status) ~
age10 + gcs.1 + pspline(iss.1, df=6) +Sex.1+ ICU.1 + ICED.1
,data=trans1, method="breslow")

anova(T.sex, T3)
```

```
## Analysis of Deviance Table
##  Cox model: response is  Surv(Tstart, Tstop, status)
##  Model 1: ~ age10 + gcs.1 + pspline(iss.1, df = 6) + Sex.1 + ICU.1 + ICED.1
##  Model 2: ~ age10 + gcs.1 + pspline(iss.1, df = 6) + ICU.1 + ICED.1
##    loglik  Chisq      Df P(>|Chi|)
## 1 -691.48
## 2 -692.65 2.3326 0.98202    0.1238
```

```r
T.ICED <- coxph(Surv(Tstart,Tstop,status) ~
age10 + gcs.1 + pspline(iss.1, df=6) + ICU.1
,data=trans1, method="breslow")

anova(T.ICED, T3)
```

```
## Analysis of Deviance Table
##  Cox model: response is  Surv(Tstart, Tstop, status)
##  Model 1: ~ age10 + gcs.1 + pspline(iss.1, df = 6) + ICU.1
##  Model 2: ~ age10 + gcs.1 + pspline(iss.1, df = 6) + ICU.1 + ICED.1
##    loglik  Chisq      Df P(>|Chi|)
## 1 -694.75
## 2 -692.65 4.2082 0.98384   0.03925 *
## ---
## Signif. codes:  0 '***' 0.001 '**' 0.01 '*' 0.05 '.' 0.1 ' ' 1
```

```r
########################################
```

Final model for time to death outcome (Table 3)

```r
##T3 is our final model
#Extract HR with 95 % CI
T3.HR=round(summary(T3)$conf.int[,c(1,3,4)], digits = 2)
T3.HR
```

```
##             exp(coef) lower .95 upper .95
## age10            1.38      1.22      1.56
## gcs.1            0.84      0.80      0.88
## ps(iss.1)3       0.59      0.08      4.41
## ps(iss.1)4       0.42      0.02      8.13
## ps(iss.1)5       0.58      0.03     12.83
## ps(iss.1)6       0.91      0.04     19.54
## ps(iss.1)7       1.12      0.05     22.97
## ps(iss.1)8       1.20      0.06     25.20
## ps(iss.1)9       2.48      0.12     50.77
## ps(iss.1)10      4.44      0.22     91.68
## ps(iss.1)11      4.13      0.19     87.83
## ps(iss.1)12      3.89      0.18     83.12
## ps(iss.1)13      4.88      0.22    106.30
## ps(iss.1)14      8.15      0.35    191.46
```

```
## ps(iss.1)15     11.38      0.42    307.85
## ps(iss.1)16      7.29      0.24    225.41
## ps(iss.1)17      2.26      0.06     79.24
## ps(iss.1)18      0.55      0.01     35.95
## ps(iss.1)19      0.13      0.00     37.86
## ICU.1            3.19      1.60      6.34
## ICED.1           1.60      1.04      2.47
```

```
#Extract p-value
T3.P=round(summary(T3)$coefficient[,6], digits = 3)
T3.P
```

```
##                      age10                       gcs.1
##                      0.000                       0.000
## pspline(iss.1, df = 6), l pspline(iss.1, df = 6), n
##                      0.000                       0.018
##                      ICU.1                      ICED.1
##                      0.001                       0.032
```

Time to discharge outcome

```
trans2=subset(sdata, trans==2)
##Cut time point of 10, 30 days
trans.2=survSplit(Surv(Tstart,Tstop, status) ~., trans2,
 cut=c(10,30),start="tstart", episode ="timegroup")
#head(trans.2)

trans.2$t10group=ifelse(trans.2$tstart<10, 1, 2)

#head(trans2)
D1 <- coxph(Surv(Tstart,Tstop,status) ~
Sex.2+ age.2 + gcs.2 + pspline(iss.2, df=5) + ICU.2 + ICED.2 +pspline(sbp.2, df=5)
,data=trans2, method="breslow")

summary(D1)
```

```
## Call:
## coxph(formula = Surv(Tstart, Tstop, status) ~ Sex.2 + age.2 +
##     gcs.2 + pspline(iss.2, df = 5) + ICU.2 + ICED.2 + pspline(sbp.2,
##     df = 5), data = trans2, method = "breslow")
##
##   n= 4997, number of events= 4810
##
##                           coef       se(coef)  se2       Chisq  DF
## Sex.2                     -0.1000604 0.0302544 0.0302422  10.94 1.00
## age.2                     -0.0025721 0.0008646 0.0008621   8.85 1.00
## gcs.2                      0.0363149 0.0100376 0.0100232  13.09 1.00
## pspline(iss.2, df = 5), l -0.0330627 0.0032123 0.0031804 105.94 1.00
## pspline(iss.2, df = 5), n                                 57.35 4.05
## ICU.2                     -0.6536389 0.0597629 0.0595672 119.62 1.00
## ICED.2                    -0.2360730 0.0427064 0.0426506  30.56 1.00
## pspline(sbp.2, df = 5), l -0.0003649 0.0005274 0.0005271   0.48 1.00
## pspline(sbp.2, df = 5), n                                 17.39 4.07
##                           p
```

```
## Sex.2                      9.4e-04
## age.2                      2.9e-03
## gcs.2                      3.0e-04
## pspline(iss.2, df = 5), l 0.0e+00
## pspline(iss.2, df = 5), n 1.1e-11
## ICU.2                      0.0e+00
## ICED.2                     3.2e-08
## pspline(sbp.2, df = 5), l 4.9e-01
## pspline(sbp.2, df = 5), n 1.7e-03
##
##              exp(coef) exp(-coef) lower .95 upper .95
## Sex.2           0.9048     1.1052   0.85269    0.9601
## age.2           0.9974     1.0026   0.99574    0.9991
## gcs.2           1.0370     0.9643   1.01678    1.0576
## ps(iss.2)3      0.9252     1.0809   0.60150    1.4230
## ps(iss.2)4      0.6791     1.4726   0.41604    1.1083
## ps(iss.2)5      0.4583     2.1819   0.29364    0.7154
## ps(iss.2)6      0.5473     1.8273   0.34439    0.8696
## ps(iss.2)7      0.5186     1.9283   0.32086    0.8382
## ps(iss.2)8      0.4289     2.3318   0.26366    0.6976
## ps(iss.2)9      0.3701     2.7016   0.22190    0.6174
## ps(iss.2)10     0.3202     3.1228   0.18383    0.5578
## ps(iss.2)11     0.2912     3.4345   0.15312    0.5537
## ps(iss.2)12     0.2543     3.9329   0.11450    0.5646
## ps(iss.2)13     0.2081     4.8054   0.07719    0.5610
## ps(iss.2)14     0.1646     6.0746   0.04808    0.5637
## ps(iss.2)15     0.1297     7.7101   0.02634    0.6386
## ps(iss.2)16     0.1024     9.7659   0.01206    0.8697
## ICU.2           0.5201     1.9225   0.46266    0.5848
## ICED.2          0.7897     1.2663   0.72631    0.8587
## ps(sbp.2)3      1.0804     0.9256   0.57949    2.0142
## ps(sbp.2)4      1.1690     0.8555   0.40134    3.4047
## ps(sbp.2)5      1.2762     0.7836   0.32581    4.9986
## ps(sbp.2)6      1.4134     0.7075   0.30642    6.5194
## ps(sbp.2)7      1.5544     0.6433   0.31923    7.5690
## ps(sbp.2)8      1.6830     0.5942   0.34697    8.1639
## ps(sbp.2)9      1.8883     0.5296   0.39189    9.0987
## ps(sbp.2)10     1.9396     0.5156   0.40223    9.3534
## ps(sbp.2)11     1.7781     0.5624   0.36845    8.5813
## ps(sbp.2)12     1.6620     0.6017   0.34383    8.0337
## ps(sbp.2)13     1.5594     0.6413   0.32170    7.5589
## ps(sbp.2)14     1.5089     0.6627   0.30687    7.4191
## ps(sbp.2)15     1.4596     0.6851   0.27571    7.7270
## ps(sbp.2)16     1.4097     0.7094   0.21775    9.1267
##
## Iterations: 7 outer, 19 Newton-Raphson
##      Theta= 0.9465612
##      Theta= 0.9654675
## Degrees of freedom for terms= 1.0 1.0 1.0 5.0 1.0 1.0 5.1
## Concordance= 0.646  (se = 0.005 )
## Likelihood ratio test= 910.2  on 15.1 df,   p=0
```

```
zp.D1=cox.zph(D1)
zp.D1
```

```
##                  rho   chisq        p
## Sex.2       -0.04152   8.237 4.11e-03
## age.2        0.01023   0.475 4.90e-01
## gcs.2       -0.00982   0.480 4.89e-01
## ps(iss.2)3   0.01923   0.355 5.51e-01
## ps(iss.2)4   0.03224   1.928 1.65e-01
## ps(iss.2)5   0.05358   4.801 2.84e-02
## ps(iss.2)6   0.02208   0.783 3.76e-01
## ps(iss.2)7   0.02634   1.208 2.72e-01
## ps(iss.2)8   0.04274   3.505 6.12e-02
## ps(iss.2)9   0.04744   4.565 3.26e-02
## ps(iss.2)10  0.04759   5.146 2.33e-02
## ps(iss.2)11  0.04529   5.710 1.69e-02
## ps(iss.2)12  0.03857   4.261 3.90e-02
## ps(iss.2)13  0.02889   2.376 1.23e-01
## ps(iss.2)14  0.01900   1.101 2.94e-01
## ps(iss.2)15  0.01183   0.432 5.11e-01
## ps(iss.2)16  0.00728   0.145 7.04e-01
## ICU.2        0.07063  24.613 7.01e-07
## ICED.2       0.00588   0.165 6.85e-01
## ps(sbp.2)3  -0.01165   0.189 6.64e-01
## ps(sbp.2)4  -0.01167   0.251 6.16e-01
## ps(sbp.2)5  -0.01174   0.318 5.73e-01
## ps(sbp.2)6  -0.01183   0.380 5.38e-01
## ps(sbp.2)7  -0.01231   0.458 4.99e-01
## ps(sbp.2)8  -0.01417   0.631 4.27e-01
## ps(sbp.2)9  -0.01526   0.726 3.94e-01
## ps(sbp.2)10 -0.01498   0.697 4.04e-01
## ps(sbp.2)11 -0.01466   0.668 4.14e-01
## ps(sbp.2)12 -0.01429   0.635 4.25e-01
## ps(sbp.2)13 -0.01475   0.678 4.10e-01
## ps(sbp.2)14 -0.01774   0.985 3.21e-01
## ps(sbp.2)15 -0.02078   1.324 2.50e-01
## ps(sbp.2)16 -0.02301   1.450 2.29e-01
## GLOBAL            NA 121.610 4.50e-12
```

```
#plot(zp.D1, resid=F)

summary(stepAIC(D1, direction="both"))
```

```
## Start:  AIC=72058.88
## Surv(Tstart, Tstop, status) ~ Sex.2 + age.2 + gcs.2 + pspline(iss.2,
##     df = 5) + ICU.2 + ICED.2 + pspline(sbp.2, df = 5)
##
##                                  Df   AIC
## <none>                              72059
## - age.2                     0.99728 72065
## - Sex.2                     0.99851 72068
## - pspline(sbp.2, df = 5) 5.06240 72068
## - gcs.2                     0.99510 72071
## - ICED.2                    0.99988 72087
## - ICU.2                     0.99613 72190
## - pspline(iss.2, df = 5) 5.03602 72220
```

```
## Call:
```

```
## coxph(formula = Surv(Tstart, Tstop, status) ~ Sex.2 + age.2 +
##     gcs.2 + pspline(iss.2, df = 5) + ICU.2 + ICED.2 + pspline(sbp.2,
##     df = 5), data = trans2, method = "breslow")
##
##   n= 4997, number of events= 4810
##
##                           coef       se(coef)  se2       Chisq  DF
## Sex.2                     -0.1000604 0.0302544 0.0302422  10.94 1.00
## age.2                     -0.0025721 0.0008646 0.0008621   8.85 1.00
## gcs.2                      0.0363149 0.0100376 0.0100232  13.09 1.00
## pspline(iss.2, df = 5), l -0.0330627 0.0032123 0.0031804 105.94 1.00
## pspline(iss.2, df = 5), n                               57.35 4.05
## ICU.2                     -0.6536389 0.0597629 0.0595672 119.62 1.00
## ICED.2                    -0.2360730 0.0427064 0.0426506  30.56 1.00
## pspline(sbp.2, df = 5), l -0.0003649 0.0005274 0.0005271   0.48 1.00
## pspline(sbp.2, df = 5), n                               17.39 4.07
##                           p
## Sex.2                     9.4e-04
## age.2                     2.9e-03
## gcs.2                     3.0e-04
## pspline(iss.2, df = 5), l 0.0e+00
## pspline(iss.2, df = 5), n 1.1e-11
## ICU.2                     0.0e+00
## ICED.2                    3.2e-08
## pspline(sbp.2, df = 5), l 4.9e-01
## pspline(sbp.2, df = 5), n 1.7e-03
##
##              exp(coef) exp(-coef) lower .95 upper .95
## Sex.2           0.9048     1.1052   0.85269    0.9601
## age.2           0.9974     1.0026   0.99574    0.9991
## gcs.2           1.0370     0.9643   1.01678    1.0576
## ps(iss.2)3      0.9252     1.0809   0.60150    1.4230
## ps(iss.2)4      0.6791     1.4726   0.41604    1.1083
## ps(iss.2)5      0.4583     2.1819   0.29364    0.7154
## ps(iss.2)6      0.5473     1.8273   0.34439    0.8696
## ps(iss.2)7      0.5186     1.9283   0.32086    0.8382
## ps(iss.2)8      0.4289     2.3318   0.26366    0.6976
## ps(iss.2)9      0.3701     2.7016   0.22190    0.6174
## ps(iss.2)10     0.3202     3.1228   0.18383    0.5578
## ps(iss.2)11     0.2912     3.4345   0.15312    0.5537
## ps(iss.2)12     0.2543     3.9329   0.11450    0.5646
## ps(iss.2)13     0.2081     4.8054   0.07719    0.5610
## ps(iss.2)14     0.1646     6.0746   0.04808    0.5637
## ps(iss.2)15     0.1297     7.7101   0.02634    0.6386
## ps(iss.2)16     0.1024     9.7659   0.01206    0.8697
## ICU.2           0.5201     1.9225   0.46266    0.5848
## ICED.2          0.7897     1.2663   0.72631    0.8587
## ps(sbp.2)3      1.0804     0.9256   0.57949    2.0142
## ps(sbp.2)4      1.1690     0.8555   0.40134    3.4047
## ps(sbp.2)5      1.2762     0.7836   0.32581    4.9986
## ps(sbp.2)6      1.4134     0.7075   0.30642    6.5194
## ps(sbp.2)7      1.5544     0.6433   0.31923    7.5690
## ps(sbp.2)8      1.6830     0.5942   0.34697    8.1639
## ps(sbp.2)9      1.8883     0.5296   0.39189    9.0987
```

```
## ps(sbp.2)10    1.9396     0.5156   0.40223    9.3534
## ps(sbp.2)11    1.7781     0.5624   0.36845    8.5813
## ps(sbp.2)12    1.6620     0.6017   0.34383    8.0337
## ps(sbp.2)13    1.5594     0.6413   0.32170    7.5589
## ps(sbp.2)14    1.5089     0.6627   0.30687    7.4191
## ps(sbp.2)15    1.4596     0.6851   0.27571    7.7270
## ps(sbp.2)16    1.4097     0.7094   0.21775    9.1267
##
## Iterations: 7 outer, 19 Newton-Raphson
##      Theta= 0.9465612
##      Theta= 0.9654675
## Degrees of freedom for terms= 1.0 1.0 1.0 5.0 1.0 1.0 5.1
## Concordance= 0.646  (se = 0.005 )
## Likelihood ratio test= 910.2  on 15.1 df,   p=0
```

```
D4=coxph(formula = Surv(Tstart, Tstop, status) ~ Sex.2 + age.2 +
    gcs.2 + pspline(iss.2, df = 5) + ICU.2 + ICED.2 + pspline(sbp.2,
    df = 5), data = trans2, method = "breslow")

summary(D4)
```

```
## Call:
## coxph(formula = Surv(Tstart, Tstop, status) ~ Sex.2 + age.2 +
##     gcs.2 + pspline(iss.2, df = 5) + ICU.2 + ICED.2 + pspline(sbp.2,
##     df = 5), data = trans2, method = "breslow")
##
##   n= 4997, number of events= 4810
##
##                            coef       se(coef)  se2       Chisq  DF
## Sex.2                      -0.1000604 0.0302544 0.0302422  10.94 1.00
## age.2                      -0.0025721 0.0008646 0.0008621   8.85 1.00
## gcs.2                       0.0363149 0.0100376 0.0100232  13.09 1.00
## pspline(iss.2, df = 5), l -0.0330627 0.0032123 0.0031804 105.94 1.00
## pspline(iss.2, df = 5), n                                  57.35 4.05
## ICU.2                      -0.6536389 0.0597629 0.0595672 119.62 1.00
## ICED.2                     -0.2360730 0.0427064 0.0426506  30.56 1.00
## pspline(sbp.2, df = 5), l -0.0003649 0.0005274 0.0005271   0.48 1.00
## pspline(sbp.2, df = 5), n                                  17.39 4.07
##                            p
## Sex.2                      9.4e-04
## age.2                      2.9e-03
## gcs.2                      3.0e-04
## pspline(iss.2, df = 5), l 0.0e+00
## pspline(iss.2, df = 5), n 1.1e-11
## ICU.2                      0.0e+00
## ICED.2                     3.2e-08
## pspline(sbp.2, df = 5), l 4.9e-01
## pspline(sbp.2, df = 5), n 1.7e-03
##
##              exp(coef) exp(-coef) lower .95 upper .95
## Sex.2           0.9048     1.1052   0.85269    0.9601
## age.2           0.9974     1.0026   0.99574    0.9991
## gcs.2           1.0370     0.9643   1.01678    1.0576
## ps(iss.2)3      0.9252     1.0809   0.60150    1.4230
## ps(iss.2)4      0.6791     1.4726   0.41604    1.1083
```

```
## ps(iss.2)5     0.4583     2.1819   0.29364    0.7154
## ps(iss.2)6     0.5473     1.8273   0.34439    0.8696
## ps(iss.2)7     0.5186     1.9283   0.32086    0.8382
## ps(iss.2)8     0.4289     2.3318   0.26366    0.6976
## ps(iss.2)9     0.3701     2.7016   0.22190    0.6174
## ps(iss.2)10    0.3202     3.1228   0.18383    0.5578
## ps(iss.2)11    0.2912     3.4345   0.15312    0.5537
## ps(iss.2)12    0.2543     3.9329   0.11450    0.5646
## ps(iss.2)13    0.2081     4.8054   0.07719    0.5610
## ps(iss.2)14    0.1646     6.0746   0.04808    0.5637
## ps(iss.2)15    0.1297     7.7101   0.02634    0.6386
## ps(iss.2)16    0.1024     9.7659   0.01206    0.8697
## ICU.2          0.5201     1.9225   0.46266    0.5848
## ICED.2         0.7897     1.2663   0.72631    0.8587
## ps(sbp.2)3     1.0804     0.9256   0.57949    2.0142
## ps(sbp.2)4     1.1690     0.8555   0.40134    3.4047
## ps(sbp.2)5     1.2762     0.7836   0.32581    4.9986
## ps(sbp.2)6     1.4134     0.7075   0.30642    6.5194
## ps(sbp.2)7     1.5544     0.6433   0.31923    7.5690
## ps(sbp.2)8     1.6830     0.5942   0.34697    8.1639
## ps(sbp.2)9     1.8883     0.5296   0.39189    9.0987
## ps(sbp.2)10    1.9396     0.5156   0.40223    9.3534
## ps(sbp.2)11    1.7781     0.5624   0.36845    8.5813
## ps(sbp.2)12    1.6620     0.6017   0.34383    8.0337
## ps(sbp.2)13    1.5594     0.6413   0.32170    7.5589
## ps(sbp.2)14    1.5089     0.6627   0.30687    7.4191
## ps(sbp.2)15    1.4596     0.6851   0.27571    7.7270
## ps(sbp.2)16    1.4097     0.7094   0.21775    9.1267
##
## Iterations: 7 outer, 19 Newton-Raphson
##      Theta= 0.9465612
##      Theta= 0.9654675
## Degrees of freedom for terms= 1.0 1.0 1.0 5.0 1.0 1.0 5.1
## Concordance= 0.646  (se = 0.005 )
## Likelihood ratio test= 910.2  on 15.1 df,   p=0
```

```
zp.D4=cox.zph(D4)
zp.D4
```

```
##                   rho   chisq        p
## Sex.2        -0.04152   8.237 4.11e-03
## age.2         0.01023   0.475 4.90e-01
## gcs.2        -0.00982   0.480 4.89e-01
## ps(iss.2)3    0.01923   0.355 5.51e-01
## ps(iss.2)4    0.03224   1.928 1.65e-01
## ps(iss.2)5    0.05358   4.801 2.84e-02
## ps(iss.2)6    0.02208   0.783 3.76e-01
## ps(iss.2)7    0.02634   1.208 2.72e-01
## ps(iss.2)8    0.04274   3.505 6.12e-02
## ps(iss.2)9    0.04744   4.565 3.26e-02
## ps(iss.2)10   0.04759   5.146 2.33e-02
## ps(iss.2)11   0.04529   5.710 1.69e-02
## ps(iss.2)12   0.03857   4.261 3.90e-02
## ps(iss.2)13   0.02889   2.376 1.23e-01
## ps(iss.2)14   0.01900   1.101 2.94e-01
```

```
## ps(iss.2)15  0.01183   0.432 5.11e-01
## ps(iss.2)16  0.00728   0.145 7.04e-01
## ICU.2        0.07063  24.613 7.01e-07
## ICED.2       0.00588   0.165 6.85e-01
## ps(sbp.2)3  -0.01165   0.189 6.64e-01
## ps(sbp.2)4  -0.01167   0.251 6.16e-01
## ps(sbp.2)5  -0.01174   0.318 5.73e-01
## ps(sbp.2)6  -0.01183   0.380 5.38e-01
## ps(sbp.2)7  -0.01231   0.458 4.99e-01
## ps(sbp.2)8  -0.01417   0.631 4.27e-01
## ps(sbp.2)9  -0.01526   0.726 3.94e-01
## ps(sbp.2)10 -0.01498   0.697 4.04e-01
## ps(sbp.2)11 -0.01466   0.668 4.14e-01
## ps(sbp.2)12 -0.01429   0.635 4.25e-01
## ps(sbp.2)13 -0.01475   0.678 4.10e-01
## ps(sbp.2)14 -0.01774   0.985 3.21e-01
## ps(sbp.2)15 -0.02078   1.324 2.50e-01
## ps(sbp.2)16 -0.02301   1.450 2.29e-01
## GLOBAL            NA 121.610 4.50e-12
```

```r
#########################################
D2 <- coxph(Surv(tstart,Tstop,status) ~
Sex.2:strata(t10group)+ age.2 + gcs.2 + pspline(iss.2, df=6) + ICU.2:strata(timegroup) + ICED.2 +psplin[illegible]
,data=trans.2, method="breslow")

summary(D2)
```

```
## Call:
## coxph(formula = Surv(tstart, Tstop, status) ~ Sex.2:strata(t10group) +
##     age.2 + gcs.2 + pspline(iss.2, df = 6) + ICU.2:strata(timegroup) +
##     ICED.2 + pspline(sbp.2, df = 5), data = trans.2, method = "breslow")
##
##   n= 6823, number of events= 4810
##
##                              coef       se(coef)  se2       Chisq  DF
## age.2                        -0.0024138 0.0008695 0.0008660   7.71 1.00
## gcs.2                         0.0364511 0.0100545 0.0100359  13.14 1.00
## pspline(iss.2, df = 6), l -0.0335370 0.0032156 0.0031939 108.78 1.00
## pspline(iss.2, df = 6), n                                  56.76 5.06
## ICED.2                       -0.2344071 0.0428085 0.0427521  29.98 1.00
## pspline(sbp.2, df = 5), l -0.0003684 0.0005275 0.0005272   0.49 1.00
## pspline(sbp.2, df = 5), n                                  18.75 4.07
## Sex.2:strata(t10group)t10 -0.0249478 0.0361212 0.0361158   0.48 1.00
## Sex.2:strata(t10group)t10 -0.2403755 0.0524453 0.0524208  21.01 1.00
## ICU.2:strata(timegroup)ti -0.9468894 0.0835755 0.0834834 128.36 1.00
## ICU.2:strata(timegroup)ti -0.4984600 0.0793111 0.0791563  39.50 1.00
## ICU.2:strata(timegroup)ti -0.1168711 0.1474939 0.1472307   0.63 1.00
##                              p
## age.2                        5.5e-03
## gcs.2                        2.9e-04
## pspline(iss.2, df = 6), l 0.0e+00
## pspline(iss.2, df = 6), n 6.1e-11
## ICED.2                       4.4e-08
## pspline(sbp.2, df = 5), l 4.9e-01
## pspline(sbp.2, df = 5), n 9.4e-04
```

```
## Sex.2:strata(t10group)t10 4.9e-01
## Sex.2:strata(t10group)t10 4.6e-06
## ICU.2:strata(timegroup)ti 0.0e+00
## ICU.2:strata(timegroup)ti 3.3e-10
## ICU.2:strata(timegroup)ti 4.3e-01
##
##                            exp(coef) exp(-coef) lower .95 upper .95
## age.2                         0.9976     1.0024   0.99589    0.9993
## gcs.2                         1.0371     0.9642   1.01689    1.0578
## ps(iss.2)3                    1.0518     0.9507   0.67696    1.6343
## ps(iss.2)4                    0.9306     1.0746   0.56463    1.5338
## ps(iss.2)5                    0.6155     1.6248   0.38439    0.9855
## ps(iss.2)6                    0.6137     1.6296   0.38020    0.9905
## ps(iss.2)7                    0.7026     1.4233   0.43261    1.1410
## ps(iss.2)8                    0.6136     1.6298   0.37238    1.0110
## ps(iss.2)9                    0.5262     1.9006   0.31777    0.8712
## ps(iss.2)10                   0.4696     2.1297   0.27943    0.7891
## ps(iss.2)11                   0.4095     2.4418   0.23498    0.7138
## ps(iss.2)12                   0.3620     2.7622   0.19996    0.6555
## ps(iss.2)13                   0.3511     2.8486   0.17701    0.6962
## ps(iss.2)14                   0.3238     3.0884   0.14002    0.7488
## ps(iss.2)15                   0.2742     3.6468   0.09953    0.7555
## ps(iss.2)16                   0.2195     4.5563   0.06760    0.7126
## ps(iss.2)17                   0.1716     5.8261   0.04336    0.6794
## ps(iss.2)18                   0.1349     7.4122   0.02422    0.7516
## ps(iss.2)19                   0.1064     9.3995   0.01106    1.0235
## ICED.2                        0.7910     1.2642   0.72738    0.8603
## ps(sbp.2)3                    1.0673     0.9370   0.56844    2.0039
## ps(sbp.2)4                    1.1416     0.8759   0.38627    3.3741
## ps(sbp.2)5                    1.2376     0.8080   0.30920    4.9533
## ps(sbp.2)6                    1.3727     0.7285   0.28997    6.4980
## ps(sbp.2)7                    1.5149     0.6601   0.30285    7.5777
## ps(sbp.2)8                    1.6480     0.6068   0.33080    8.2098
## ps(sbp.2)9                    1.8648     0.5363   0.37685    9.2277
## ps(sbp.2)10                   1.9106     0.5234   0.38580    9.4619
## ps(sbp.2)11                   1.7434     0.5736   0.35177    8.6402
## ps(sbp.2)12                   1.6437     0.6084   0.33112    8.1592
## ps(sbp.2)13                   1.5308     0.6532   0.30753    7.6200
## ps(sbp.2)14                   1.4428     0.6931   0.28584    7.2822
## ps(sbp.2)15                   1.3549     0.7381   0.24962    7.3538
## ps(sbp.2)16                   1.2703     0.7872   0.19179    8.4141
## Sex.2:strata(t10group)t10    0.9754     1.0253   0.90870    1.0469
## Sex.2:strata(t10group)t10    0.7863     1.2717   0.70952    0.8715
## ICU.2:strata(timegroup)ti    0.3879     2.5777   0.32933    0.4570
## ICU.2:strata(timegroup)ti    0.6075     1.6462   0.52001    0.7096
## ICU.2:strata(timegroup)ti    0.8897     1.1240   0.66634    1.1879
##
## Iterations: 7 outer, 20 Newton-Raphson
##      Theta= 0.9434908
##      Theta= 0.9651809
## Degrees of freedom for terms= 1.0 1.0 6.1 1.0 5.1 2.0 3.0
## Concordance= 0.649  (se = 0.006 )
## Likelihood ratio test= 963.2  on 19.11 df,   p=0
```

```r
zp.D2=cox.zph(D2)
zp.D2
```

```
##                                             rho   chisq        p
## age.2                                   0.00831  0.3146 0.574875
## gcs.2                                  -0.01224  0.7465 0.387585
## ps(iss.2)3                              0.00918  0.0847 0.771072
## ps(iss.2)4                              0.01639  0.6045 0.436884
## ps(iss.2)5                              0.03637  2.7069 0.099917
## ps(iss.2)6                              0.03282  1.9471 0.162904
## ps(iss.2)7                              0.00268  0.0147 0.903501
## ps(iss.2)8                              0.01970  0.8108 0.367888
## ps(iss.2)9                              0.03411  2.5181 0.112550
## ps(iss.2)10                             0.03404  2.6211 0.105449
## ps(iss.2)11                             0.03289  2.4836 0.115038
## ps(iss.2)12                             0.03503  3.1701 0.074997
## ps(iss.2)13                             0.03795  4.5119 0.033659
## ps(iss.2)14                             0.03577  3.9633 0.046501
## ps(iss.2)15                             0.03008  2.5235 0.112165
## ps(iss.2)16                             0.02186  1.3574 0.243988
## ps(iss.2)17                             0.01370  0.6065 0.436126
## ps(iss.2)18                             0.00805  0.2204 0.638724
## ps(iss.2)19                             0.00445  0.0605 0.805653
## ICED.2                                  0.00366  0.0636 0.800910
## ps(sbp.2)3                             -0.01345  0.2650 0.606679
## ps(sbp.2)4                             -0.01345  0.3507 0.553734
## ps(sbp.2)5                             -0.01347  0.4377 0.508211
## ps(sbp.2)6                             -0.01346  0.5106 0.474897
## ps(sbp.2)7                             -0.01389  0.6025 0.437613
## ps(sbp.2)8                             -0.01567  0.7972 0.371932
## ps(sbp.2)9                             -0.01656  0.8826 0.347497
## ps(sbp.2)10                            -0.01632  0.8542 0.355373
## ps(sbp.2)11                            -0.01603  0.8256 0.363541
## ps(sbp.2)12                            -0.01548  0.7698 0.380285
## ps(sbp.2)13                            -0.01603  0.8258 0.363502
## ps(sbp.2)14                            -0.01920  1.1905 0.275235
## ps(sbp.2)15                            -0.02242  1.5875 0.207689
## ps(sbp.2)16                            -0.02484  1.7359 0.187664
## Sex.2:strata(t10group)t10group=1       -0.00659  0.2087 0.647766
## Sex.2:strata(t10group)t10group=2        0.00930  0.4076 0.523179
## ICU.2:strata(timegroup)timegroup=1      0.02701  3.8324 0.050271
## ICU.2:strata(timegroup)timegroup=2      0.00653  0.2003 0.654474
## ICU.2:strata(timegroup)timegroup=3     -0.00173  0.0138 0.906617
## GLOBAL                                       NA 78.3039 0.000191
```

```r
#plot(zp.D2, resid=F)

trans.2$age10=trans.2$age.2/10
########################################
D3 <- coxph(Surv(tstart,Tstop,status) ~
pspline(iss.2, df=7) +pspline(sbp.2, df=5)+Sex.2:strata(t10group)+ age10 + gcs.2 +  ICU.2:strata(timegr
,data=trans.2, method="breslow")

summary(D3)
```

```
## Call:
## coxph(formula = Surv(tstart, Tstop, status) ~ pspline(iss.2,
##     df = 7) + pspline(sbp.2, df = 5) + Sex.2:strata(t10group) +
##     age10 + gcs.2 + ICU.2:strata(timegroup) + ICED.2, data = trans.2,
##     method = "breslow")
##
##   n= 6823, number of events= 4810
##
##                           coef       se(coef)  se2       Chisq  DF
## pspline(iss.2, df = 7), l -0.0335535 0.0032151 0.0031948 108.92 1.00
## pspline(iss.2, df = 7), n                                 60.80 6.06
## pspline(sbp.2, df = 5), l -0.0003985 0.0005277 0.0005274   0.57 1.00
## pspline(sbp.2, df = 5), n                                 18.75 4.07
## age10                     -0.0231601 0.0087248 0.0086886   7.05 1.00
## gcs.2                      0.0364277 0.0100614 0.0100372  13.11 1.00
## ICED.2                    -0.2313601 0.0428561 0.0428108  29.14 1.00
## Sex.2:strata(t10group)t10 -0.0247544 0.0361235 0.0361194   0.47 1.00
## Sex.2:strata(t10group)t10 -0.2396904 0.0524569 0.0524349  20.88 1.00
## ICU.2:strata(timegroup)ti -0.9507160 0.0836504 0.0835602 129.17 1.00
## ICU.2:strata(timegroup)ti -0.5043263 0.0793958 0.0792629  40.35 1.00
## ICU.2:strata(timegroup)ti -0.1248732 0.1476309 0.1473531   0.72 1.00
##                           p
## pspline(iss.2, df = 7), l 0.0e+00
## pspline(iss.2, df = 7), n 3.3e-11
## pspline(sbp.2, df = 5), l 4.5e-01
## pspline(sbp.2, df = 5), n 9.4e-04
## age10                     7.9e-03
## gcs.2                     2.9e-04
## ICED.2                    6.7e-08
## Sex.2:strata(t10group)t10 4.9e-01
## Sex.2:strata(t10group)t10 4.9e-06
## ICU.2:strata(timegroup)ti 0.0e+00
## ICU.2:strata(timegroup)ti 2.1e-10
## ICU.2:strata(timegroup)ti 4.0e-01
##
##                           exp(coef) exp(-coef) lower .95 upper .95
## ps(iss.2)3                   1.1334     0.8823   0.70952    1.8104
## ps(iss.2)4                   1.1047     0.9052   0.66334    1.8396
## ps(iss.2)5                   0.7742     1.2916   0.46917    1.2777
## ps(iss.2)6                   0.6615     1.5116   0.40301    1.0859
## ps(iss.2)7                   0.7438     1.3444   0.44753    1.2363
## ps(iss.2)8                   0.7949     1.2579   0.47893    1.3195
## ps(iss.2)9                   0.6712     1.4900   0.39790    1.1321
## ps(iss.2)10                  0.5939     1.6839   0.34963    1.0087
## ps(iss.2)11                  0.5511     1.8145   0.32396    0.9375
## ps(iss.2)12                  0.5051     1.9800   0.28619    0.8913
## ps(iss.2)13                  0.4305     2.3231   0.23648    0.7835
## ps(iss.2)14                  0.3985     2.5093   0.21143    0.7512
## ps(iss.2)15                  0.4138     2.4164   0.20041    0.8546
## ps(iss.2)16                  0.4042     2.4740   0.16723    0.9770
## ps(iss.2)17                  0.3569     2.8019   0.12230    1.0415
## ps(iss.2)18                  0.2923     3.4214   0.08558    0.9982
## ps(iss.2)19                  0.2297     4.3538   0.05979    0.8823
## ps(iss.2)20                  0.1790     5.5867   0.03985    0.8039
```

```
## ps(iss.2)21                  0.1417     7.0589   0.02260    0.8881
## ps(iss.2)22                  0.1126     8.8801   0.01011    1.2544
## ps(sbp.2)3                   1.0686     0.9358   0.56931    2.0056
## ps(sbp.2)4                   1.1443     0.8739   0.38747    3.3795
## ps(sbp.2)5                   1.2416     0.8054   0.31057    4.9634
## ps(sbp.2)6                   1.3774     0.7260   0.29144    6.5098
## ps(sbp.2)7                   1.5179     0.6588   0.30402    7.5788
## ps(sbp.2)8                   1.6479     0.6068   0.33141    8.1940
## ps(sbp.2)9                   1.8634     0.5366   0.37727    9.2037
## ps(sbp.2)10                  1.9097     0.5236   0.38634    9.4397
## ps(sbp.2)11                  1.7401     0.5747   0.35177    8.6080
## ps(sbp.2)12                  1.6401     0.6097   0.33101    8.1260
## ps(sbp.2)13                  1.5267     0.6550   0.30728    7.5855
## ps(sbp.2)14                  1.4365     0.6961   0.28512    7.2374
## ps(sbp.2)15                  1.3467     0.7426   0.24854    7.2968
## ps(sbp.2)16                  1.2606     0.7933   0.19060    8.3368
## age10                        0.9771     1.0234   0.96054    0.9940
## gcs.2                        1.0371     0.9642   1.01685    1.0578
## ICED.2                       0.7935     1.2603   0.72953    0.8630
## Sex.2:strata(t10group)t10    0.9755     1.0251   0.90887    1.0471
## Sex.2:strata(t10group)t10    0.7869     1.2709   0.70999    0.8721
## ICU.2:strata(timegroup)ti    0.3865     2.5876   0.32802    0.4553
## ICU.2:strata(timegroup)ti    0.6039     1.6559   0.51688    0.7056
## ICU.2:strata(timegroup)ti    0.8826     1.1330   0.66085    1.1788
##
## Iterations: 7 outer, 20 Newton-Raphson
##      Theta= 0.9349269
##      Theta= 0.9651878
## Degrees of freedom for terms= 7.1 5.1 1.0 1.0 1.0 2.0 3.0
## Concordance= 0.649  (se = 0.006 )
## Likelihood ratio test= 966.2  on 20.1 df,   p=0
```

```r
zp.D3=cox.zph(D3)
zp.D3
```

```
##                                          rho    chisq        p
## ps(iss.2)3                          0.006291 3.40e-02 8.54e-01
## ps(iss.2)4                          0.011667 3.07e-01 5.80e-01
## ps(iss.2)5                          0.024654 1.33e+00 2.49e-01
## ps(iss.2)6                          0.035944 2.34e+00 1.26e-01
## ps(iss.2)7                          0.019042 7.26e-01 3.94e-01
## ps(iss.2)8                         -0.004670 4.68e-02 8.29e-01
## ps(iss.2)9                          0.020509 8.97e-01 3.43e-01
## ps(iss.2)10                         0.033737 2.45e+00 1.18e-01
## ps(iss.2)11                         0.029056 1.93e+00 1.64e-01
## ps(iss.2)12                         0.025731 1.57e+00 2.11e-01
## ps(iss.2)13                         0.025503 1.53e+00 2.17e-01
## ps(iss.2)14                         0.031082 2.57e+00 1.09e-01
## ps(iss.2)15                         0.036225 4.35e+00 3.71e-02
## ps(iss.2)16                         0.035453 4.13e+00 4.21e-02
## ps(iss.2)17                         0.031736 2.77e+00 9.63e-02
## ps(iss.2)18                         0.025524 1.60e+00 2.06e-01
## ps(iss.2)19                         0.017380 8.16e-01 3.66e-01
## ps(iss.2)20                         0.010116 3.46e-01 5.56e-01
## ps(iss.2)21                         0.005618 1.19e-01 7.30e-01
```

```
## ps(iss.2)22                          0.002850 2.78e-02 8.68e-01
## ps(sbp.2)3                          -0.013579 2.69e-01 6.04e-01
## ps(sbp.2)4                          -0.013585 3.56e-01 5.51e-01
## ps(sbp.2)5                          -0.013600 4.44e-01 5.05e-01
## ps(sbp.2)6                          -0.013572 5.17e-01 4.72e-01
## ps(sbp.2)7                          -0.013975 6.07e-01 4.36e-01
## ps(sbp.2)8                          -0.015747 8.02e-01 3.71e-01
## ps(sbp.2)9                          -0.016634 8.87e-01 3.46e-01
## ps(sbp.2)10                         -0.016406 8.60e-01 3.54e-01
## ps(sbp.2)11                         -0.016108 8.30e-01 3.62e-01
## ps(sbp.2)12                         -0.015532 7.72e-01 3.80e-01
## ps(sbp.2)13                         -0.016097 8.29e-01 3.62e-01
## ps(sbp.2)14                         -0.019268 1.19e+00 2.75e-01
## ps(sbp.2)15                         -0.022468 1.59e+00 2.08e-01
## ps(sbp.2)16                         -0.024852 1.73e+00 1.88e-01
## age10                                0.008195 3.07e-01 5.80e-01
## gcs.2                               -0.012824 8.19e-01 3.65e-01
## ICED.2                               0.002822 3.79e-02 8.46e-01
## Sex.2:strata(t10group)t10group=1    -0.006496 2.03e-01 6.52e-01
## Sex.2:strata(t10group)t10group=2     0.009258 4.04e-01 5.25e-01
## ICU.2:strata(timegroup)timegroup=1   0.028073 4.14e+00 4.18e-02
## ICU.2:strata(timegroup)timegroup=2   0.008039 3.05e-01 5.81e-01
## ICU.2:strata(timegroup)timegroup=3 -0.000065 1.94e-05 9.96e-01
## GLOBAL                                     NA 8.55e+01 8.49e-05
```

```
D4 <- coxph(Surv(tstart,Tstop,status) ~
pspline(iss.2, df=5) +pspline(sbp.2, df=5)+Sex.2:strata(t10group)+ age10 + gcs.2 +  ICU.2:strata(timegro
,data=trans.2, method="breslow")

summary(D4)
```

```
## Call:
## coxph(formula = Surv(tstart, Tstop, status) ~ pspline(iss.2,
##     df = 5) + pspline(sbp.2, df = 5) + Sex.2:strata(t10group) +
##     age10 + gcs.2 + ICU.2:strata(timegroup) + ICED.2, data = trans.2,
##     method = "breslow")
##
##   n= 6823, number of events= 4810
##
##                           coef       se(coef)  se2       Chisq  DF
## pspline(iss.2, df = 5), l -0.0334615 0.0032278 0.0031961 107.47 1.00
## pspline(iss.2, df = 5), n                                 50.84 4.05
## pspline(sbp.2, df = 5), l -0.0003244 0.0005274 0.0005271   0.38 1.00
## pspline(sbp.2, df = 5), n                                 18.72 4.07
## age10                     -0.0259495 0.0086514 0.0086275   9.00 1.00
## gcs.2                      0.0364649 0.0100510 0.0100349  13.16 1.00
## ICED.2                    -0.2400509 0.0427356 0.0426801  31.55 1.00
## Sex.2:strata(t10group)t10 -0.0250996 0.0361185 0.0361115   0.48 1.00
## Sex.2:strata(t10group)t10 -0.2423031 0.0524347 0.0524105  21.35 1.00
## ICU.2:strata(timegroup)ti -0.9396961 0.0834877 0.0833771 126.69 1.00
## ICU.2:strata(timegroup)ti -0.4873267 0.0791992 0.0790030  37.86 1.00
## ICU.2:strata(timegroup)ti -0.1015462 0.1473613 0.1471371   0.47 1.00
##                           p
## pspline(iss.2, df = 5), l 0.0e+00
## pspline(iss.2, df = 5), n 2.6e-10
```

```
## pspline(sbp.2, df = 5), l 5.4e-01
## pspline(sbp.2, df = 5), n 9.5e-04
## age10                      2.7e-03
## gcs.2                      2.9e-04
## ICED.2                     1.9e-08
## Sex.2:strata(t10group)t10 4.9e-01
## Sex.2:strata(t10group)t10 3.8e-06
## ICU.2:strata(timegroup)ti 0.0e+00
## ICU.2:strata(timegroup)ti 7.6e-10
## ICU.2:strata(timegroup)ti 4.9e-01
##
##                            exp(coef) exp(-coef) lower .95 upper .95
## ps(iss.2)3                   0.91383     1.0943   0.59332    1.4075
## ps(iss.2)4                   0.67150     1.4892   0.41077    1.0977
## ps(iss.2)5                   0.46228     2.1632   0.29574    0.7226
## ps(iss.2)6                   0.54577     1.8323   0.34292    0.8686
## ps(iss.2)7                   0.51246     1.9514   0.31655    0.8296
## ps(iss.2)8                   0.42122     2.3741   0.25853    0.6863
## ps(iss.2)9                   0.35547     2.8132   0.21273    0.5940
## ps(iss.2)10                  0.30065     3.3262   0.17230    0.5246
## ps(iss.2)11                  0.27191     3.6777   0.14237    0.5193
## ps(iss.2)12                  0.23634     4.2311   0.10582    0.5279
## ps(iss.2)13                  0.19151     5.2215   0.07080    0.5180
## ps(iss.2)14                  0.14954     6.6873   0.04371    0.5116
## ps(iss.2)15                  0.11627     8.6010   0.02368    0.5708
## ps(iss.2)16                  0.09061    11.0368   0.01069    0.7678
## ps(sbp.2)3                   1.06707     0.9371   0.56829    2.0036
## ps(sbp.2)4                   1.14119     0.8763   0.38606    3.3734
## ps(sbp.2)5                   1.23694     0.8084   0.30894    4.9525
## ps(sbp.2)6                   1.37227     0.7287   0.28970    6.5003
## ps(sbp.2)7                   1.51777     0.6589   0.30315    7.5990
## ps(sbp.2)8                   1.65658     0.6037   0.33221    8.2605
## ps(sbp.2)9                   1.87581     0.5331   0.37872    9.2909
## ps(sbp.2)10                  1.92123     0.5205   0.38758    9.5236
## ps(sbp.2)11                  1.75630     0.5694   0.35404    8.7126
## ps(sbp.2)12                  1.65709     0.6035   0.33351    8.2335
## ps(sbp.2)13                  1.54469     0.6474   0.31003    7.6963
## ps(sbp.2)14                  1.45969     0.6851   0.28892    7.3745
## ps(sbp.2)15                  1.37431     0.7276   0.25299    7.4657
## ps(sbp.2)16                  1.29186     0.7741   0.19491    8.5625
## age10                        0.97438     1.0263   0.95800    0.9910
## gcs.2                        1.03714     0.9642   1.01691    1.0578
## ICED.2                       0.78659     1.2713   0.72339    0.8553
## Sex.2:strata(t10group)t10    0.97521     1.0254   0.90856    1.0468
## Sex.2:strata(t10group)t10    0.78482     1.2742   0.70817    0.8698
## ICU.2:strata(timegroup)ti    0.39075     2.5592   0.33176    0.4602
## ICU.2:strata(timegroup)ti    0.61427     1.6280   0.52595    0.7174
## ICU.2:strata(timegroup)ti    0.90344     1.1069   0.67681    1.2060
##
## Iterations: 7 outer, 20 Newton-Raphson
##      Theta= 0.9462261
##      Theta= 0.9651839
## Degrees of freedom for terms= 5.0 5.1 1.0 1.0 1.0 2.0 3.0
## Concordance= 0.647  (se = 0.006 )
```

```
## Likelihood ratio test= 956.1  on 18.09 df,   p=0
```

```r
zp.D4=cox.zph(D4)
zp.D4
```

```
##                                          rho   chisq        p
## ps(iss.2)3                           0.01693  0.2764 0.599041
## ps(iss.2)4                           0.03033  1.7135 0.190533
## ps(iss.2)5                           0.05260  4.6486 0.031078
## ps(iss.2)6                           0.02061  0.6868 0.407266
## ps(iss.2)7                           0.02499  1.0944 0.295502
## ps(iss.2)8                           0.04147  3.3170 0.068565
## ps(iss.2)9                           0.04575  4.2523 0.039198
## ps(iss.2)10                          0.04623  4.8669 0.027377
## ps(iss.2)11                          0.04493  5.6886 0.017076
## ps(iss.2)12                          0.03893  4.4017 0.035903
## ps(iss.2)13                          0.02964  2.5245 0.112092
## ps(iss.2)14                          0.01982  1.2002 0.273287
## ps(iss.2)15                          0.01255  0.4845 0.486408
## ps(iss.2)16                          0.00789  0.1692 0.680856
## ps(sbp.2)3                          -0.01319  0.2558 0.613016
## ps(sbp.2)4                          -0.01320  0.3384 0.560769
## ps(sbp.2)5                          -0.01321  0.4221 0.515902
## ps(sbp.2)6                          -0.01319  0.4918 0.483145
## ps(sbp.2)7                          -0.01363  0.5814 0.445766
## ps(sbp.2)8                          -0.01541  0.7734 0.379174
## ps(sbp.2)9                          -0.01631  0.8593 0.353932
## ps(sbp.2)10                         -0.01606  0.8292 0.362492
## ps(sbp.2)11                         -0.01579  0.8030 0.370189
## ps(sbp.2)12                         -0.01528  0.7522 0.385789
## ps(sbp.2)13                         -0.01582  0.8061 0.369272
## ps(sbp.2)14                         -0.01900  1.1678 0.279862
## ps(sbp.2)15                         -0.02224  1.5648 0.210963
## ps(sbp.2)16                         -0.02468  1.7174 0.190033
## age10                                0.00951  0.4116 0.521137
## gcs.2                               -0.01187  0.7027 0.401881
## ICED.2                               0.00529  0.1330 0.715349
## Sex.2:strata(t10group)t10group=1   -0.00672  0.2174 0.641015
## Sex.2:strata(t10group)t10group=2    0.00942  0.4181 0.517906
## ICU.2:strata(timegroup)timegroup=1  0.02533  3.3644 0.066619
## ICU.2:strata(timegroup)timegroup=2  0.00402  0.0758 0.783052
## ICU.2:strata(timegroup)timegroup=3 -0.00384  0.0679 0.794457
## GLOBAL                                   NA 68.6535 0.000839
```

```r
anova(D3, D4)
```

```
## Analysis of Deviance Table
##  Cox model: response is  Surv(tstart, Tstop, status)
##  Model 1: ~ pspline(iss.2, df = 7) + pspline(sbp.2, df = 5) + Sex.2:strata(t10group) + age10 + gcs.2
##  Model 2: ~ pspline(iss.2, df = 5) + pspline(sbp.2, df = 5) + Sex.2:strata(t10group) + age10 + gcs.2
##   loglik  Chisq     Df P(>|Chi|)
## 1 -35986
## 2 -35991 10.065 2.0107  0.006604 **
## ---
## Signif. codes:  0 '***' 0.001 '**' 0.01 '*' 0.05 '.' 0.1 ' ' 1
```

Final model for time to discharge outcome (Table 3)

```
#Extract HR with 95 % CI
D3.HR=round(summary(D3)$conf.int[,c(1,3,4)], digits = 2)

D3.HR
```

```
##                             exp(coef) lower .95 upper .95
## ps(iss.2)3                       1.13      0.71      1.81
## ps(iss.2)4                       1.10      0.66      1.84
## ps(iss.2)5                       0.77      0.47      1.28
## ps(iss.2)6                       0.66      0.40      1.09
## ps(iss.2)7                       0.74      0.45      1.24
## ps(iss.2)8                       0.79      0.48      1.32
## ps(iss.2)9                       0.67      0.40      1.13
## ps(iss.2)10                      0.59      0.35      1.01
## ps(iss.2)11                      0.55      0.32      0.94
## ps(iss.2)12                      0.51      0.29      0.89
## ps(iss.2)13                      0.43      0.24      0.78
## ps(iss.2)14                      0.40      0.21      0.75
## ps(iss.2)15                      0.41      0.20      0.85
## ps(iss.2)16                      0.40      0.17      0.98
## ps(iss.2)17                      0.36      0.12      1.04
## ps(iss.2)18                      0.29      0.09      1.00
## ps(iss.2)19                      0.23      0.06      0.88
## ps(iss.2)20                      0.18      0.04      0.80
## ps(iss.2)21                      0.14      0.02      0.89
## ps(iss.2)22                      0.11      0.01      1.25
## ps(sbp.2)3                       1.07      0.57      2.01
## ps(sbp.2)4                       1.14      0.39      3.38
## ps(sbp.2)5                       1.24      0.31      4.96
## ps(sbp.2)6                       1.38      0.29      6.51
## ps(sbp.2)7                       1.52      0.30      7.58
## ps(sbp.2)8                       1.65      0.33      8.19
## ps(sbp.2)9                       1.86      0.38      9.20
## ps(sbp.2)10                      1.91      0.39      9.44
## ps(sbp.2)11                      1.74      0.35      8.61
## ps(sbp.2)12                      1.64      0.33      8.13
## ps(sbp.2)13                      1.53      0.31      7.59
## ps(sbp.2)14                      1.44      0.29      7.24
## ps(sbp.2)15                      1.35      0.25      7.30
## ps(sbp.2)16                      1.26      0.19      8.34
## age10                            0.98      0.96      0.99
## gcs.2                            1.04      1.02      1.06
## ICED.2                           0.79      0.73      0.86
## Sex.2:strata(t10group)t10        0.98      0.91      1.05
## Sex.2:strata(t10group)t10        0.79      0.71      0.87
## ICU.2:strata(timegroup)ti        0.39      0.33      0.46
## ICU.2:strata(timegroup)ti        0.60      0.52      0.71
## ICU.2:strata(timegroup)ti        0.88      0.66      1.18
```

```
#Extract p-value
D3.P=round(summary(D3)$coefficient[,6], digits = 3)
D3.P
```

```
## pspline(iss.2, df = 7), l pspline(iss.2, df = 7), n
##                     0.000                     0.000
## pspline(sbp.2, df = 5), l pspline(sbp.2, df = 5), n
##                     0.450                     0.001
##                     age10                     gcs.2
##                     0.008                     0.000
##                    ICED.2 Sex.2:strata(t10group)t10
##                     0.000                     0.493
## Sex.2:strata(t10group)t10 ICU.2:strata(timegroup)ti
##                     0.000                     0.000
## ICU.2:strata(timegroup)ti ICU.2:strata(timegroup)ti
##                     0.000                     0.398
```

Nonlinear plot (Figure 2)

```
par(mfrow=c(2,2))
##Model T2, pspine(iss.1, df=5)
##Nonlinear plot for ISS
Ptemp <- termplot(T3, term=3, se=T, plot=F)
attributes(Ptemp)
```

```
## $constant
## [1] -0.8106209
##
## $names
## [1] "iss.1"
```

```
Ptemp$iss.1[1:4,]
```

```
##   x           y        se
## 1 1 -0.09974553 0.6118633
## 2 2 -0.20886982 0.4509239
## 3 3 -0.29488468 0.3206855
## 4 4 -0.34829493 0.2284124
```

```
ISSterm <- Ptemp$iss.1  # this will be a data frame
str(ISSterm)
```

```
## 'data.frame':    37 obs. of  3 variables:
##  $ x : num  1 2 3 4 5 6 8 9 10 11 ...
##  $ y : num  -0.0997 -0.2089 -0.2949 -0.3483 -0.3597 ...
##  $ se: num  0.612 0.451 0.321 0.228 0.173 ...
```

```
summary(ISSterm)
```

```
##        x               y                  se
##  Min.   : 1.00   Min.   :-0.35967   Min.   :0.1404
##  1st Qu.:11.00   1st Qu.: 0.04862   1st Qu.:0.3032
##  Median :21.00   Median : 0.73116   Median :0.3484
##  Mean   :23.35   Mean   : 0.93596   Mean   :0.4215
##  3rd Qu.:34.00   3rd Qu.: 1.79870   3rd Qu.:0.4429
##  Max.   :57.00   Max.   : 2.69919   Max.   :1.5318
```

```
Center <- with(ISSterm, y[x==9])
```

```r
summary(Center)
```

```
##      Min.   1st Qu.    Median      Mean   3rd Qu.      Max.
## -0.06058 -0.06058 -0.06058 -0.06058 -0.06058 -0.06058
```

```r
Ytemp <- ISSterm$y + outer(ISSterm$se, c(0, -1.96, 1.96), '*')
matplot(ISSterm$x, exp(Ytemp - Center), xlim = c(1,25),ylim = c(0, 10), type='l', lty=c(1,2,2), col=c("
xlab="ISS scores", ylab="Relative death rate", main="Admission to death")

##Model D3, pspine(iss.1, df=5)
##Nonlinear plot for ISS
Ptemp <- termplot(D3, term=1, se=T, plot=F)
attributes(Ptemp)
```

```
## $constant
## [1] 0.5142787
##
## $names
## [1] "iss.2"
```

```r
Ptemp$iss.2[1:4,]
```

```
##   x         y         se
## 1 1 0.3158766 0.07384982
## 2 2 0.3230931 0.04751973
## 3 3 0.3087838 0.02918676
## 4 4 0.2670066 0.02235862
```

```r
ISSterm <- Ptemp$iss.2  # this will be a data frame
str(ISSterm)
```

```
## 'data.frame':    37 obs. of  3 variables:
##  $ x : num  1 2 3 4 5 6 8 9 10 11 ...
##  $ y : num  0.316 0.323 0.309 0.267 0.195 ...
##  $ se: num  0.0738 0.0475 0.0292 0.0224 0.0254 ...
```

```r
summary(ISSterm)
```

```
##        x               y                  se
##  Min.   : 1.00   Min.   :-1.73774   Min.   :0.01866
##  1st Qu.:11.00   1st Qu.:-0.66877   1st Qu.:0.04752
##  Median :21.00   Median :-0.23327   Median :0.07789
##  Mean   :23.35   Mean   :-0.35013   Mean   :0.16694
##  3rd Qu.:34.00   3rd Qu.:-0.07317   3rd Qu.:0.17169
##  Max.   :57.00   Max.   : 0.32309   Max.   :0.90242
```

```r
Center <- with(ISSterm, y[x==9])

summary(Center)
```

```
##    Min. 1st Qu.  Median    Mean 3rd Qu.    Max.
## -0.1188 -0.1188 -0.1188 -0.1188 -0.1188 -0.1188
```

```r
Ytemp <- ISSterm$y + outer(ISSterm$se, c(0, -1.96, 1.96), '*')
matplot(ISSterm$x, exp(Ytemp - Center), xlim = c(1,25),ylim = c(0, 2), type='l', lty=c(1,2,2), col=c("o
xlab="ISS scores", ylab="Relative discharge rate", main="Admission to Discharge")

##Nonlinear plot for SBP
```

```
Ptemp <- termplot(D3, term=2, se=T, plot=F)
attributes(Ptemp)
```

```
## $constant
## [1] 0.5142787
##
## $names
## [1] "sbp.2"
```

```
Ptemp$sbp.2[1:4,]
```

```
##    x          y        se
## 1  0 -0.4999795 0.5675971
## 2 46 -0.3285372 0.2174328
## 3 52 -0.3004203 0.1865762
## 4 54 -0.2908610 0.1770744
```

```
SBPterm <- Ptemp$sbp.2  # this will be a data frame
str(SBPterm)
```

```
## 'data.frame':    184 obs. of  3 variables:
##  $ x : num  0 46 52 54 55 56 58 60 62 65 ...
##  $ y : num  -0.5 -0.329 -0.3 -0.291 -0.286 ...
##  $ se: num  0.568 0.217 0.187 0.177 0.172 ...
```

```
summary(SBPterm)
```

```
##        x               y                   se
##  Min.   :  0.0   Min.   :-0.499980   Min.   :0.01750
##  1st Qu.:102.8   1st Qu.:-0.167045   1st Qu.:0.02294
##  Median :148.5   Median :-0.083591   Median :0.03917
##  Mean   :148.3   Mean   :-0.087031   Mean   :0.06357
##  3rd Qu.:194.2   3rd Qu.: 0.006033   3rd Qu.:0.07898
##  Max.   :252.0   Max.   : 0.065756   Max.   :0.56760
```

```
Center <- with(SBPterm, y[x==150])

summary(Center)
```

```
##    Min. 1st Qu.  Median    Mean 3rd Qu.    Max.
## 0.05468 0.05468 0.05468 0.05468 0.05468 0.05468
```

```
Ytemp <- SBPterm$y + outer(SBPterm$se, c(0, -1.96, 1.96), '*')
matplot(SBPterm$x, exp(Ytemp - Center), xlim = c(70,180),ylim = c(0, 2), type='l', lty=c(1,2,2), col=c(
xlab="SBP (mmHg)", ylab="Relative discharge rate", main="Admission to Discharge")
```

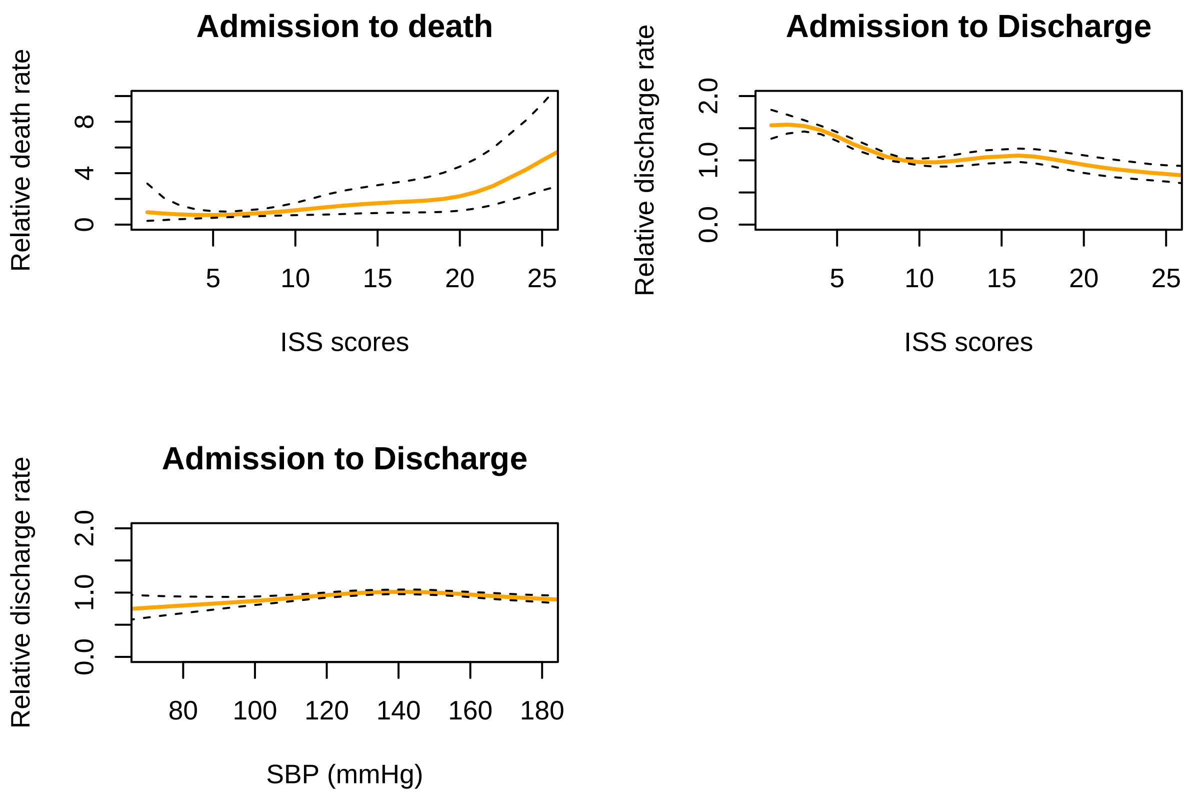

ISS scores stratified by sex

```
par(mfrow=c(1,1))
boxplot(trauma$iss~trauma$Sex, ylim=c(0,80))
```

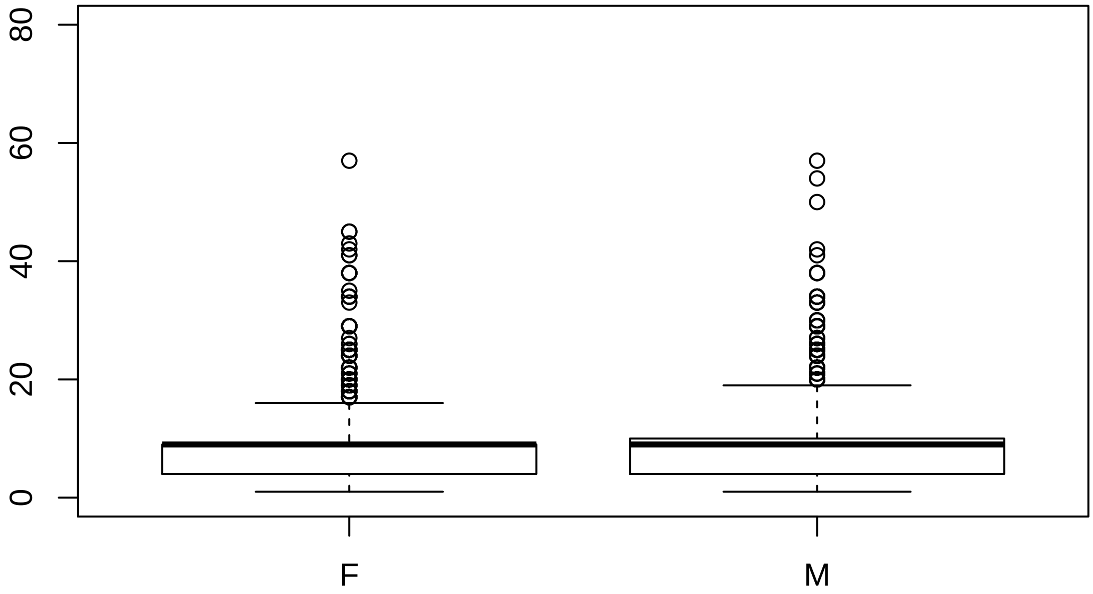


```
summaryBy(iss~Sex, data=trauma,  FUN=c(mean,median,IQR, range))
```

```
##   Sex iss.FUN1 iss.FUN2 iss.FUN3 iss.FUN4 iss.FUN5
## 1   F 8.402952        9        5        1       57
```

```
## 2   M 9.275263        9        6        1       57
```

```r
wilcox.test(iss~Sex, data=trauma)
```

```
##
##  Wilcoxon rank sum test with continuity correction
##
## data:  iss by Sex
## W = 2918600, p-value = 0.0005217
## alternative hypothesis: true location shift is not equal to 0
```

Log-linear model for conditional independence

```r
library(vcd)
```

```
## Loading required package: grid
```

```r
Data=subset(trauma, select=c("Death", "ICED_23", "ISS_3", "iss"))

Data$ICED <- factor(Data$ICED_23, labels = c("0/1", "2/3"))
#table(Data$ICED)
#table(Data$ICED_23)

Data$ISS <- factor(Data$ISS_3, labels = c("<16", "16-25", ">=25"))
#table(Data$ISS_3)
#table(Data$ISS)

Data$Mortality <- factor(Data$Death, labels = c("No", "Yes"))
#table(Data$Death)
#table(Data$Mortality)

alz <- xtabs(~ ICED + Mortality + ISS, data = Data)
alz
```

```
## , , ISS = <16
##
##      Mortality
## ICED    No  Yes
##   0/1 3526    8
##   2/3  681   16
##
## , , ISS = 16-25
##
##      Mortality
## ICED    No  Yes
##   0/1  406   11
##   2/3   85    9
##
## , , ISS = >=25
##
##      Mortality
## ICED    No  Yes
##   0/1  147   55
##   2/3   38   15
```

```
###################################################### Mosaic plot conditional on ISS
####################################################
rseed <- 1071
set.seed(rseed)
cotabplot(~ ICED + Mortality | ISS, data = alz, panel = cotab_coindep, n = 5000)
```

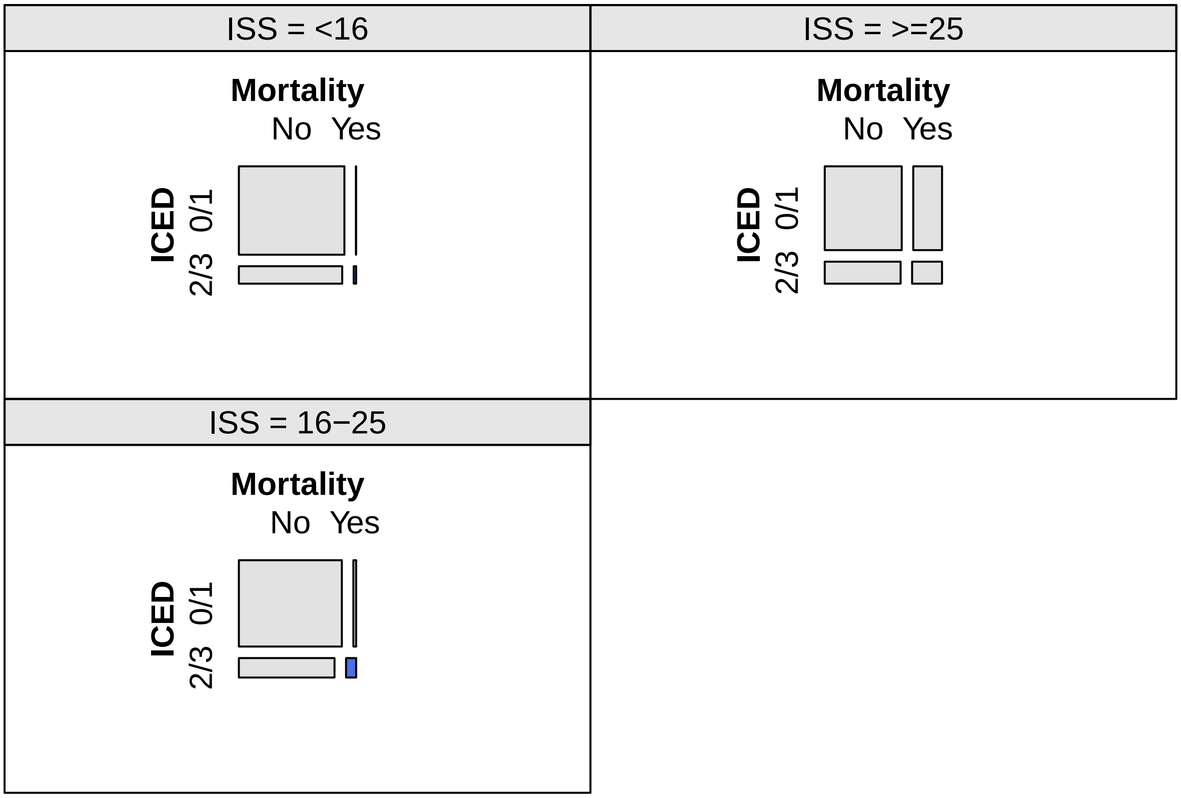


```
######################################################Check indepedence test for log-linear model using ##c
#####################################
ss <- function(x) sum(x^2)
set.seed(rseed)
coindep_test(alz, 3, n = 5000)
```

```
##
##  Permutation test for conditional independence
##
## data:  alz
## f(x) = 6.0583, p-value < 2.2e-16
```

```
set.seed(rseed)
coindep_test(alz, 3, n = 5000, indepfun = ss)
```

```
##
##  Permutation test for conditional independence
##
## data:  alz
## f(x) = 44.193, p-value < 2.2e-16
```

```
set.seed(rseed)
coindep_test(alz, 3, n = 5000, indepfun = ss, aggfun = sum)
```

```
##
##  Permutation test for conditional independence
##
## data:  alz
## f(x) = 54.032, p-value < 2.2e-16
```

```
##############################################
Split <- c(T,F, T)
##HCL shading, Figure 3
mosaic(alz, expected = ~ (Mortality + ICED)*ISS,gp = shading_hcl, split_vertical = Split)
```

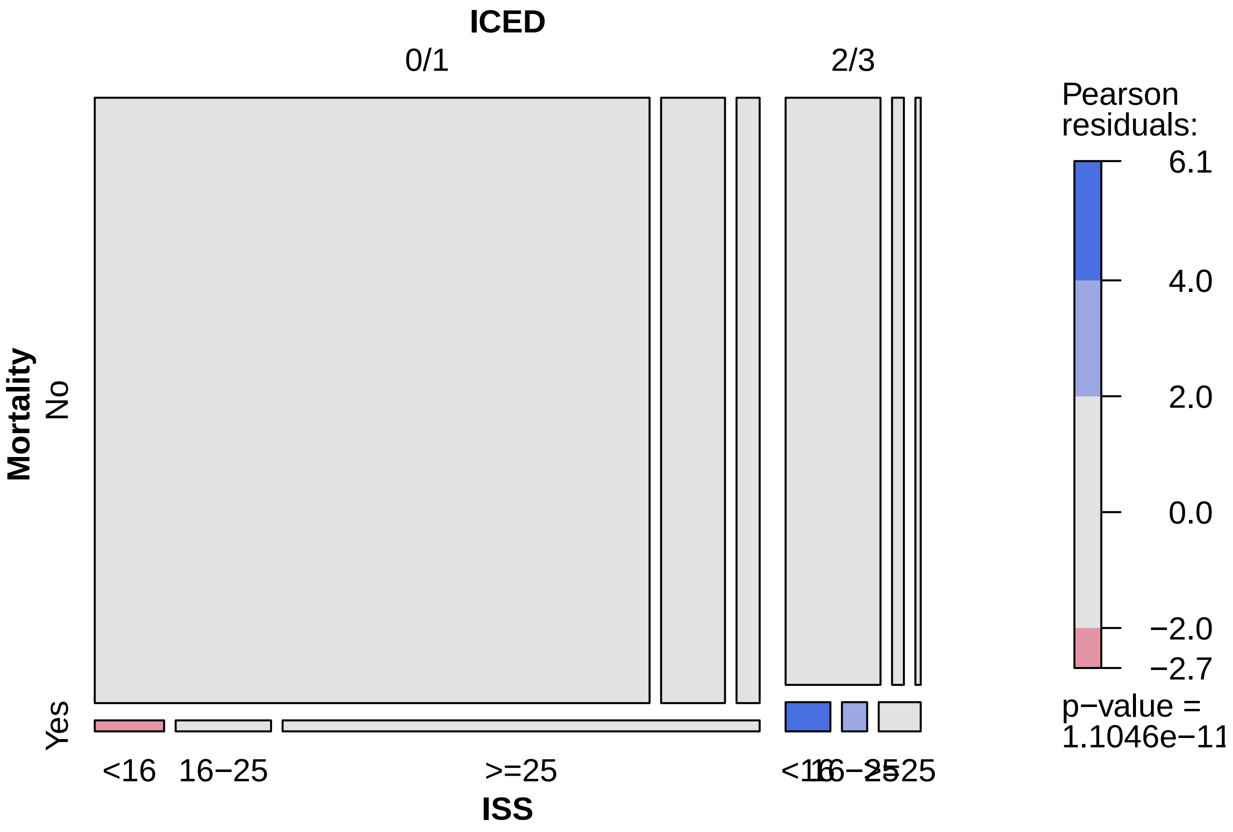


```
##Friendly shading
mosaic(alz, expected = ~ (Mortality + ICED)*ISS,gp = shading_Friendly, split_vertical = Split)
```

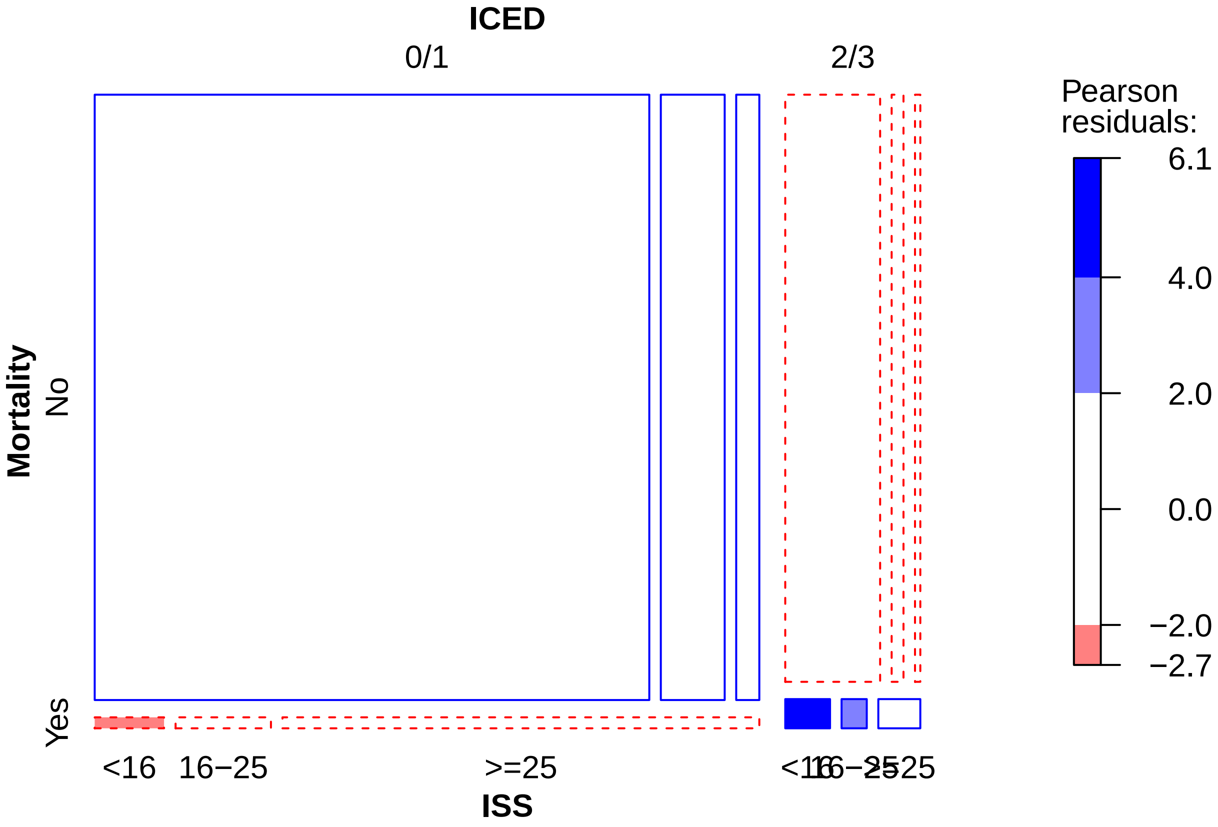
ICED
0/1
2/3
Mortality
No
Yes
<16
16−25
>=25
ISS
Pearson
residuals:
6.1
4.0
2.0
0.0
−2.0
−2.7
