# Supplementary material for: Impact of comorbidities on the prognoses of trauma patients: Analysis of a hospital-based trauma registry database
Source: PLoS One. 2018 Mar 20;13(3):e0194749. doi: 10.1371/journal.pone.0194749 (PMC5860791; doi:10.1371/journal.pone.0194749)
Supplement: S1 Table — (DOCX) [file pone.0194749.s004.docx]

**Table S1. Baseline patient demographics and characteristics (N = 4997)**

| **Characteristics** | |
| --- | --- |
| Sex, *n* (%) |  |
| Male | 2761 (55.3) |
| Female | 2236 (44.7) |
| Age, years | |
| Mean (standard deviation; SD) | 59 (20) |
| Median (interquartile range; IQ) | 59 (44,75) |
| Age group, years, *n* (%) | |
| 15–54 | 2629 (52.6) |
| 55–69 | 744 (14.9) |
| 70–79 | 833 (16.7) |
| > 80 | 791 (15.8) |
| Glasgow coma scale (GCS), *n* (%) | |
| < 13 | 355 (7.1) |
| ≥ 13 | 4642 (92.9) |
| Systolic blood pressure (SBP), mmHg |  |
| Mean (SD) | 144 (29) |
| Median (IQ) | 142 (125, 163) |
| < 90 mmHg, *n* (%) | 81 (1.6) |
| ≥ 90 mmHg, *n* (%) | 4916 (98.4) |
| Respiratory rate (mean, SD) | 18.5 ±1.7 |
| Injury Severity Score (ISS), *n* (%) |  |
| < 5 | 1904 (38.1) |
| 5–15 | 2327 (46.6) |
| 16­–24 | 511 (10.2) |
| > 25 | 255 (5.1) |
| ICU admission, *n* (%) |  |
| Yes | 695 (13.9) |
| No | 4302 (86.1) |
| Comorbidity number, *n* (%) |  |
| 0 | 2483 (49.7) |
| 1 | 1180 (23.6) |
| 2 | 957 (19.2) |
| ≥ 3 | 377 (7.5) |
| Index of Coexisting Disease, *n* (%) |  |
| 0–1 | 4153 (83.1) |
| 2–3 | 844 (16.9) |

IQ = interquartile
