# Supplementary material for: Impact of comorbidities on the prognoses of trauma patients: Analysis of a hospital-based trauma registry database
Source: PLoS One. 2018 Mar 20;13(3):e0194749. doi: 10.1371/journal.pone.0194749 (PMC5860791; doi:10.1371/journal.pone.0194749)
Supplement: S2 Table — (DOCX) [file pone.0194749.s005.docx]

Data description

Sex: F=famle, M=male (categorical)

age: years (numeric)

alcohol: alcohol consumption, yes=1, n=0 (binary)

gcs: points (numeric)

GCS: GCS<13=0, GCS>=13=1 (categorical)

pr: baseline pulse rate (numeric)

sbp: baseline systolic blood pressure (numeric)

rr: baseline respiratory rate (numeric)

AIS: highest AIS region, A=abdomen, E=extremity, H=head, T=thoracic (categorical)

iss: Injury Severity Score (numeric)

ICU: ICU admission, yes=1, no=0 (binary)

ADday: length of stay, days (numeric)

Death: yes=1, no=0 (binary)

Discharge: yes=1, no=0 (binary)

Co_no: number of comorbidity (numeric)

ICED_N: class of ICED, 0,1,2,3 (categorical)

ICED_23: A=ICED 0/1, B=ICED 2/3 (categorical)

status: 0=transfer to other hospital, 1=hospital mortality, 2=alive discharge (categorical)
